# Developing affordable and efficient heating devices for enhanced live cell imaging in confocal microscopy

**DOI:** 10.3389/fpls.2024.1499831

**Published:** 2025-01-10

**Authors:** Abhishesh Bajracharya, Sampada Timilsina, Ruofan Cao, Qingrui Jiang, Berry A. Dickey, Anupa Wasti, Jing Xi, Magdalena Weingartner, Scott R. Baerson, Gregg W. Roman, Yiwei Han, Yongjian Qiu

**Affiliations:** ^1^ Department of Biology, University of Mississippi, University, MS, United States; ^2^ Department of Biomolecular Sciences, School of Pharmacy, University of Mississippi, University, MS, United States; ^3^ Department of Mechanical Engineering, University of Mississippi, University, MS, United States; ^4^ Natural Products Utilization Research Unit, U.S. Department of Agriculture, Agricultural Research Service, University, MS, United States; ^5^ Institute of Plant Sciences and Microbiology, University of Hamburg, Hamburg, Germany

**Keywords:** cost-effective, low-maintenance, confocal microscopy, live cell imaging, heat stress, heat shock protein, stress granules (SG), microheater

## Abstract

Temperature control is crucial for live cell imaging, particularly in studies involving plant responses to high ambient temperatures and thermal stress. This study presents the design, development, and testing of two cost-effective heating devices tailored for confocal microscopy applications: an aluminum heat plate and a wireless mini-heater. The aluminum heat plate, engineered to integrate seamlessly with the standard 160 mm × 110 mm microscope stage, supports temperatures up to 36°C, suitable for studies in the range of non-stressful warm temperatures (e.g., 25-27°C for *Arabidopsis thaliana*) and moderate heat stress (e.g., 30-36°C for *A. thaliana*). We also developed a wireless mini-heater that offers rapid, precise heating directly at the sample slide, with a temperature increase rate over 30 times faster than the heat plate. The wireless heater effectively maintained target temperatures up to 50°C, ideal for investigating severe heat stress and heat shock responses in plants. Both devices performed well in controlled studies, including the real-time analysis of heat shock protein accumulation and stress granule formation in *A. thaliana*. Our designs are effective and affordable, with total construction costs lower than $300. This accessibility makes them particularly valuable for small laboratories with limited funding. Future improvements could include enhanced heat uniformity, humidity control to mitigate evaporation, and more robust thermal management to minimize focus drift during extended imaging sessions. These modifications would further solidify the utility of our heating devices in live cell imaging, offering researchers reliable, budget-friendly tools for exploring plant thermal biology.

## Introduction

Temperature serves as a pivotal environmental cue that profoundly influences the viability and functionality of life across the diverse spectrum of organisms inhabiting Earth. Each organism exhibits a specific temperature range conducive to executing essential biological processes, spanning from inception to the culmination of life cycles. Consequently, environmental temperature fluctuations exert profound and far-reaching impacts on virtually every facet of growth and developmental pathways, transcending taxonomic boundaries to encompass microorganisms, fungi, plants, and animals (Ding et al., 2020; Nord and Giroud, 2020; Buckley and Kingsolver, 2021; Wang et al., 2021).

Given this fundamental role of temperature in biological systems, a closer examination at the microscopic level becomes essential to fully understand how these temperature cues affect individual cells and small-scale biological interactions (Oyama et al., 2022). This microscopic focus allows for a deeper comprehension of biological processes at a cellular level, further driving advancements in fields such as medicine, agriculture, and conservation. To achieve this, reliable temperature control systems in microscopic studies are crucial, as they enable researchers to precisely manipulate and monitor the microenvironment, thereby observing temperature-sensitive biological phenomena with high accuracy (Andersson et al., 2021).

Precise temperature regulation is particularly critical in live-cell imaging for organisms with a body temperature significantly higher than ambient temperatures, where maintaining the correct environmental conditions is necessary to observe natural cellular behaviors such as motility, division, wound healing, and calcium signaling (O’Carroll et al., 2023). By stabilizing the temperature, researchers can prevent disruptions caused by thermal fluctuations, leading to more accurate and reproducible results. Temperature control is also pivotal in studying extremophiles, which thrive under extreme temperature conditions and require specific environmental temperatures for accurate live imaging (Molinaro et al., 2022). On the other hand, the ability to induce controlled temperature changes allows researchers to study heat shock responses and other heat stress-related cellular processes. By carefully manipulating the temperature, researchers can explore how cells respond to heat shock and other thermal stresses, providing insights into cellular adaptation mechanisms (Hong et al., 2024).

Temperature control systems are also integral to plant-related microscopic research, particularly in fields studying the dynamic responses of plant cells to environmental stressors. These systems enable precise manipulation of thermal conditions essential for observing plant physiology under stress. For instance, the work by Midorikawa and Kodama explores how plant cellular structures, particularly chloroplasts in *Marchantia polymorpha*, adapt to temperature fluctuations, demonstrating significant morphological changes with temperature variations (Midorikawa and Kodama, 2024). Similarly, Buchner et al. investigate the reversible structural transformations of chloroplasts in *Ranunculus glacialis*, an alpine species that undergoes adaptation in response to cold environments (Buchner et al., 2007).

Integrated heating systems designed by major microscope manufacturers, such as Leica, Zeiss, and Olympus, exemplify high-end solutions that maintain precise control over environmental parameters, including temperature, humidity, and gas composition. While these systems offer robust performance and reliability, they are not without drawbacks. They often come with high prices and high maintenance costs, which can be prohibitive for smaller laboratories or institutions with limited budgets.

Alternatively, microfluidic approaches offer a more compact solution, enabling fine-scale temperature control directly on the microscope stage. Innovations like the polydimethylsiloxane (PDMS)-based device developed by Casquillas et al. allow for rapid temperature changes, facilitating the study of temperature-sensitive genetic mutations in model organisms such as *Schizosaccharomyces pombe* (Casquillas et al., 2011). However, the integration of microfluidic systems with standard microscopy setups can be challenging, often requiring custom-made designs that add to the overall cost and complexity of experiments.

Laser-assisted heating techniques, such as those used in the IR-LEGO system, offer precise localized heating and are particularly useful for studies requiring spatial precision in gene expression without damaging cells (Deguchi et al., 2009). Despite their precision, these systems require significant technical expertise and are generally more expensive, making them less accessible for routine laboratory use.

Open-source advanced heating stages and low-cost imaging incubators, such as VAHEAT (Icha et al., 2022), ThermoCyte (O’Carroll et al., 2023), and the Arduino-based system (Rossi et al., 2022), provide cost-effective and customizable options. These systems are designed to allow researchers to precisely control thermal environments while minimizing costs by using commonly available components. For example, the Arduino-based imaging incubator maintains cell viability over extended periods, demonstrating its utility for prolonged live-cell imaging. However, the assembly and customization of these systems often require a certain level of technical skill, which may pose a barrier for researchers with limited experience in electronics or mechanical engineering. Additionally, the cost of some devices, such as VAHEAT, has increased significantly after commercialization. While still more affordable than sophisticated instruments offered by major manufacturers, these higher costs can make them inaccessible to many researchers operating with limited budgets.

Despite the availability of the above-mentioned temperature control systems, their direct application in observing plant responses to heat stress remains limited, particularly for studying cellular dynamics such as heat shock proteins, stress granules, and processing bodies. Typically, researchers subject plants to heat treatment outside the imaging environment and subsequently analyze the cellular effects post-treatment. This approach, while applicable, does not allow for the dynamic, real-time observation of how cells react to heat stress, including the formation and function of heat shock proteins and stress granules. The high costs, technical complexities, and limited accessibility associated with existing systems have collectively restricted their application in plant thermal biology.

In addressing the challenges associated with existing temperature control systems for plant heat stress studies, our research has pioneered the development of two affordable, adaptable, and user-friendly heating devices optimized for confocal microscopy: an aluminum heat plate and a wireless mini-heater. The aluminum heat plate, designed to integrate with the standard 160 mm × 110 mm sample holder on the Leica SP8 confocal microscope, features a user-friendly design with a moderate temperature increase speed and the ability to maintain temperatures up to 36°C. This makes it suitable for a range of studies, from moderate heat stress to non-stressful heat treatments involved in thermomorphogenesis, although it exhibits some uneven temperature distribution. To complement the heat plate and address its limitations in temperature uniformity, we introduce the wireless mini-heater. This device stands out due to its efficient heat transfer capabilities, achieved through direct contact with the sample slide. Fabricated from a copper foil tape shaped into a spiral coil, the mini-heater is straightforward to use and allows for rapid and precise temperature adjustments. Its design ensures a more uniform temperature distribution at the sample, which is critical for accurately observing dynamic cellular responses. Together, these devices offer robust, adaptable, and cost-effective solutions for conducting detailed, real-time imaging of plant cellular responses to various thermal conditions.

## Results

### Design of an aluminum heat plate for live cell imaging

**Figure 1:**
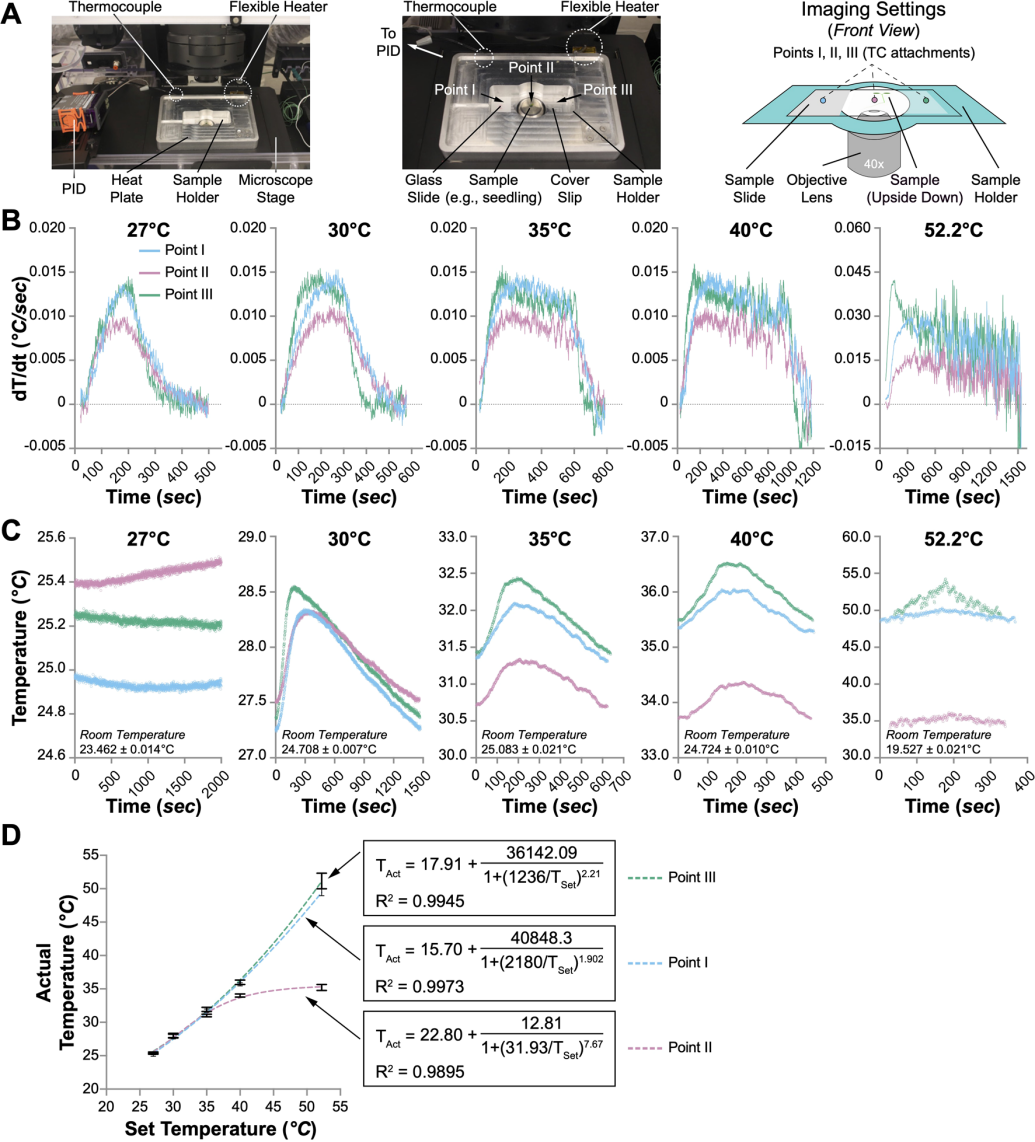
Performance of the heat plate. **(A)** The heat plate settings for microscopic usage. Only the sample holder is shown, while the rest of the heat plate is omitted in the illustration on the right panel. The sample slide is upside down, with the coverslip facing the objective lens. The temperature changes at three spots (Points I, II, and III) were monitored with thermocouples (TCs). **(B)** The speed of temperature change (dT/dt, °C/sec) at Points I, II, and III when the heat plate was set to different temperatures (27°C, 30°C, 35°C, 40°C, and 52.2°C). **(C)** Temperature fluctuations at Points I, II, and III when the heat plate was set to different temperatures. One oscillation cycle of the temperature changes when the heat plate was set to 30°C, 35°C, 40°C, and 52.2°C is shown. When the heat plate was set to 27°C, no noticeable oscillation was observed within 8 hours; therefore, 2000 seconds of temperature changes are shown instead. **(D)** The correlation between the actual and set temperatures at Points I, II, and III. Nonlinear regression (sigmoidal, 4PL) is performed to evaluate the relationship between the set temperature (T_Set_) and the actual temperature (T_Act_). The formulas and R^2^ values are shown.

We first designed and engineered an aluminum heating platform, measuring 160 mm *×* 110 mm *×* 22 mm, tailored to integrate seamlessly with the stage of the Leica SP8 confocal microscope (**Supplementary Figure 1**). The platform’s central section incorporates a recessed mount, accommodating standard microscope slides (up to 80 mm *×* 30 mm) and Petri dishes (up to 40 mm in diameter), as illustrated in **Figure 1A** and **Supplementary Figure 1**. Additionally, a circular aperture of 29 mm diameter at the core facilitates unobstructed microscopy via objective lenses.

Temperature management is achieved using a flexible heater affixed to the platform’s left side (dimensions: 80 mm *×* 25.5 mm) directly adjacent to the slide cradle. Temperature accuracy is maintained via a commercial proportional-integral-derivative (PID) controller, which is connected to a thermocouple located strategically on the right side of the platform, 22 mm from the edge, directly across from the slide holder. This arrangement—separating the heater from the temperature sensor—is designed to enhance heat distribution uniformly across the sample area (**Supplementary Figure 1**). Initial testing, involving thermocouples at six distinct points on the heating platform’s surface, revealed variations in temperature distribution. Specifically, areas proximal to the flexible heater (positions 5 and 6) exhibited frequent temperature overshoots relative to the target settings (**Supplementary Figure 2**). Conversely, zones adjacent to the thermocouples (positions 1 and 2) recorded slightly cooler temperatures than desired. Notably, the central positions near the sample chamber (positions 3 and 4) maintained temperatures closely aligned with the target, displaying minimal fluctuations (**Supplementary Figure 2**). Despite the noted temperature distribution disparities, the current design effectively ensures optimal thermal conditions near the critical sample holder area, aligning with the requirements for precise biological experimentation.

Additional functionalities of the heating platform include integrated gas flow channels within the sample area, featuring inlets and outlets, each 5 mm in diameter, to stabilize CO_2_ concentrations essential for cell culture (**Supplementary Figure 1**). A designated recess in the upper right quadrant of the platform accommodates a thermoelectric cooler, specifically a Peltier module, with dimensions not exceeding 35 mm *×* 41 mm (**Supplementary Figure 1**). This setup, if applied, allows for more rapid and precise temperature adjustments after heating phases.

### Heating performance of the aluminum heat plate

Initial assessments revealed a notable variance between the preset and actual temperatures across different areas of the heating platform (**Supplementary Figure 2**). This observation led us to evaluate the heating efficacy on biological specimens. Specifically, four-day-old Arabidopsis seedlings were prepared on a standard glass slide (75 mm *×* 25 mm) sandwiched with a coverslip (44 mm *×* 22 mm) in deionized water. Individual thermocouples were affixed at three critical points on the slide to precisely monitor and record the thermal dynamics when the heat plate was set to different temperatures (27°C, 30°C, 35°C, 40°C, and 52.2°C). Points I and III are positioned at the left and right edges of the slide, respectively, in contact with the heat plate, while Point II is centrally located directly above the objective lens aperture (**Figure 1A**).

Initial evaluations of the heating platform were conducted by assessing thermal performance at all three designated points during the preliminary heating phase, with the PID controller set to varying temperatures. It was hypothesized that the rate of temperature increase at Point II would be slower and less pronounced compared to Points I and III. This is attributed to its lack of direct contact with the heating surface, higher heat capacity of water than the glass, and the potential for enhanced airflow through the objective lens aperture at Point II. Our results confirmed this hypothesis, as the maximal temperature rise rate at Point II was only 0.01°C/sec, which is 33% slower than the rates observed at Points I and III (approximately 0.015°C/sec) under PID settings of 27°C, 30°C, 35°C, and 40°C (**Figure 1B**). Notably, although the peak rate at Point II reached 0.015°C/sec at a PID setting of 52.2°C, the peak rates at Points I and III more than doubled (**Figure 1B**). Furthermore, the highest recorded temperature at Point II consistently lagged behind those at Points I and III, with none of the points achieving the temperatures predetermined by the PID settings (**Supplementary Figure 3A**). Despite the slower temperature rise rate and the lower peak temperature at Point II, the duration required to achieve the peak temperature at all three points was similar, increasing from approximately 200 seconds at a PID setting of 27°C to nearly 1,500 seconds at 52.2°C (**Supplementary Figure 3A**).

We subsequently assessed temperature stability at all three points during the oscillation phase at various set temperatures. The temperatures recorded at these points displayed noticeable oscillations at all settings except for 27°C (**Figure 1C**). The duration of these oscillations varied, with average cycles lasting over 1440 seconds at 30°C and under 400 seconds at 52.2°C (**Supplementary Figure 3B**). Throughout a typical oscillation cycle, the temperature fluctuation between the maximum and minimum values at all points remained below 1°C (**Supplementary Figure 3C**). At set temperatures of 27°C and 30°C, the temperatures at the three points were similar. However, at settings 10°C above ambient temperature, the temperature at Point II consistently lagged behind those at Points I and III, with the discrepancy increasing at higher settings (**Figure 1C**). This pattern persisted across the settings, with actual temperatures at all points falling below those set on the PID. Further analysis involved plotting the average temperatures measured by the thermocouples at each point against the PID settings, revealing no linear correlation, particularly at Point II (**Figure 1D**). Non-linear sigmoidal data fitting for each point showed high coefficients of determination (R^2^ values of 0.9973, 0.9895, and 0.9945 for Points I, II, and III, respectively), indicating significant fidelity in the model fit. These results suggest that Points I and III—directly contacting the heating surface—are more likely to approach the set temperatures. In contrast, the maximum achievable temperature at Point II was approximately 35.6°C, irrespective of higher PID settings (**Figure 1D**).

While the aluminum heat plate was primarily tested on microscope slides, we also evaluated its performance with Petri dishes to explore broader applications. However, the heating performance on Petri dishes was suboptimal. For a 30-mm Petri dish, the center temperature reached only 32°C when the PID was set to 54.4°C (data not shown). This limitation was due to the small contact area between the Petri dish and the heat plate’s sample holder, which reduced heating efficiency. To address this, the sample holder could be redesigned to accommodate larger Petri dishes with a larger contact area. Additionally, condensation formed on the Petri dish lid during heating, impairing image quality. Although this could be addressed by adding an extra heater to warm the lid, it would increase both cost and design complexity. As a result, we opted to continue experiments using standard glass slides (75 mm × 25 mm).

### Testing the aluminum heat plate for live cell imaging

The observed temperature stability at the central sample location (Point II) confirms that our custom-designed aluminum heating platform is well-suited for live imaging applications in thermal biology. To validate this system with biological specimens, we engineered transgenic Arabidopsis lines harboring the heat-inducible marker gene *AtHSP70-4* (AT3G12580). This gene was chosen due to its robust inducibility by a broad temperature spectrum, ranging from 27°C to over 40°C (**Supplementary Figure 4A**), as evidenced by previous RNA-seq analyses conducted by our team and others (Zhang et al., 2017; Friedrich et al., 2021; Bajracharya et al., 2022). The transgene was expressed under the control of its native promoter, and the protein product was tagged at the N-terminus with a 3*×*HA-tag and a yellow fluorescent protein (YFP), creating the construct *AtHSP70-4pro::3×HA-YFP-AtHSP70-4* (abbreviated as *YFP-HSP70*). We developed over ten transgenic lines with a single insertion of this construct. Following an evaluation of YFP-HSP70 protein levels in the T3 homozygous transgenic plants, line #13 was selected for detailed confocal microscopy analysis. This line demonstrated high expression levels following moderate heat stress of 36°C for two hours (**Supplementary Figure 4B**).

We initially investigated the YFP-HSP70 fluorescence in line #13 under a controlled heat treatment using a water bath maintained at 36°C, closely mirroring the upper-temperature limit observed at Point II of the aluminum heat plate (**Figure 1D**). Five-day-old seedlings of *YFP-HSP70* were subjected to this regimen for up to 150 minutes, with specimens collected every 30 minutes for immediate confocal microscopy. Images were captured from the epidermal cells of the cotyledons and the hypocotyl cells (**Supplementary Figure 4C**). Before heating, fluorescence was minimally detectable within the cytoplasm and nuclei of both cotyledon pavement and guard cells, as well as hypocotyl cells. Upon prolonged exposure to heat, a gradual intensification of the fluorescent signal was noted in these cellular compartments, corroborating the high inducibility of *AtHSP70-4* by thermal stress as previously documented in RNA-seq analyses (Zhang et al., 2017; Friedrich et al., 2021; Bajracharya et al., 2022). The subcellular distribution pattern also aligns with the known nucleocytoplasmic localization of AtHSP70-4 (Leng et al., 2017). The effective response of *YFP-HSP70* line #13 in the water bath substantiates its suitability for subsequent analyses using the heating plate.

**Figure 2:**
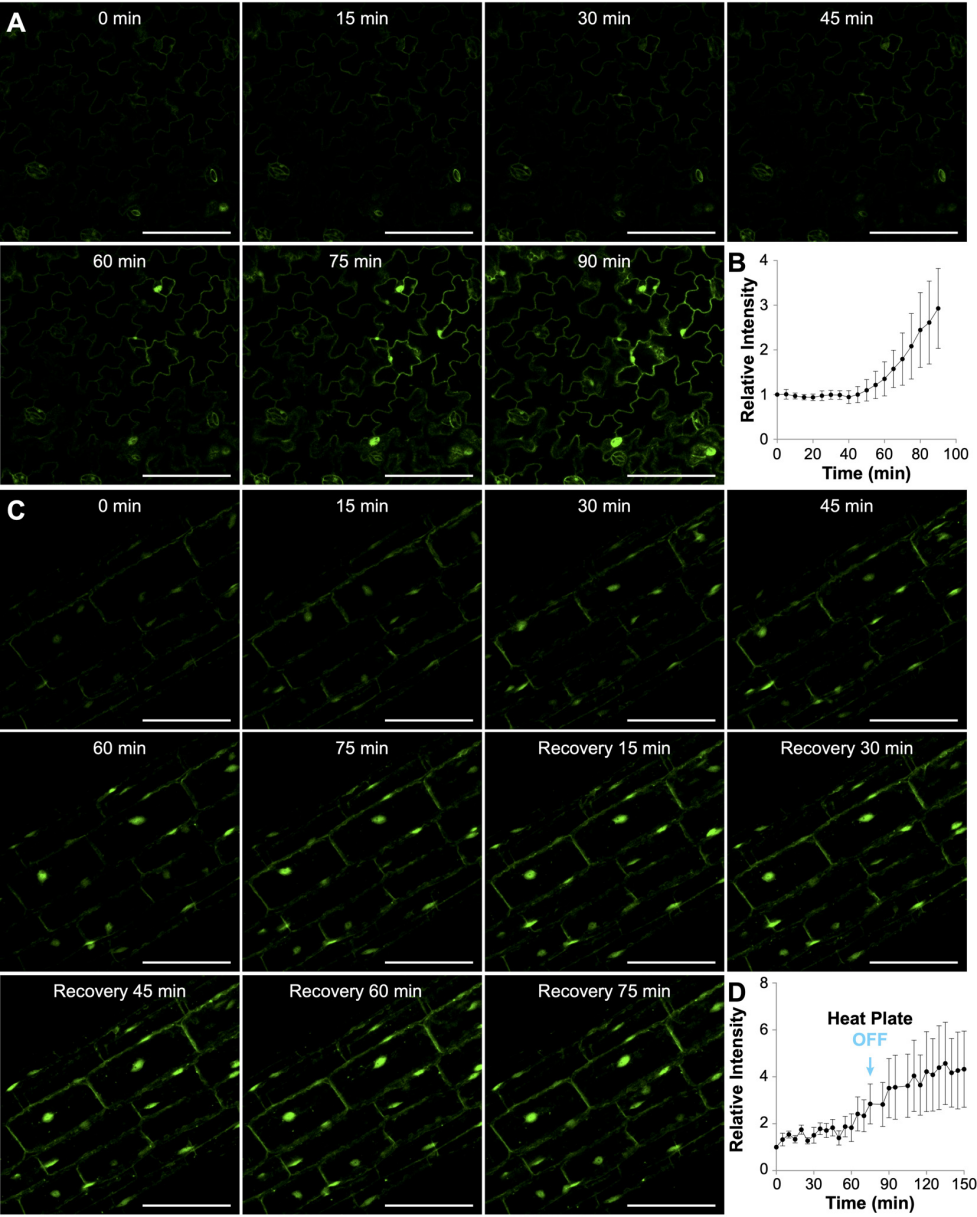
Heat-induced accumulation of AtHSP70-4 in Arabidopsis seedlings treated with the heat plate at 36°C. **(A, C)** Time-lapse confocal images showing yellow fluorescent protein (YFP) signal in cotyledon pavement cells **(A)** or hypocotyl cells **(C)** of Arabidopsis seedlings expressing *AtHSP70-4pro::YFP-AtHSP70-4*. Scale bar, 100 μm. In **(A)**, seedlings were continuously heated with the heat plate for 90 minutes, while in **(C)**, the heat plate was turned off after 75 minutes. **(B, D)** Quantification of mean fluorescence intensities of YFP-AtHSP70-4 in cotyledon pavement cells **(B)** and hypocotyl cells **(D)** over time. Fluorescence intensities were measured from 14 cotyledon pavement cells across three individual seedlings **(B)** and 37 hypocotyl cells across three individual seedlings **(D)**. Relative fluorescence intensity at each time point was normalized to the intensity at the start time (0 min).

To investigate the dynamics of YFP-HSP70 fluorescence at the cellular level, we positioned *YFP-HSP70* line #13 seedlings at Point II on the aluminum heat plate, preset to 52.2°C. Based on our prior characterization, we noted that the temperature at Point II would reach 35.6°C approximately 25 minutes after activation, followed by fluctuations between 34.4°C and 36.4°C (**Supplementary Figures 3A and 3C**). Time-lapse images were captured at 5-minute intervals, and the fluorescence intensity was quantitatively analyzed per cell. Significant YFP-HSP70 accumulation became apparent 60 minutes post-initiation in the epidermal cells of cotyledons, with marked increases observed thereafter in both the cytoplasm and nucleus (**Figure 2A** and **Supplementary Video 1**). Quantitative analysis indicated that the fluorescence intensity in pavement cells increased threefold following a 90-minute exposure to heat (**Figure 2B**). The increase of YFP-HSP70 intensity seems to be delayed and less prominent compared with that in the water bath treatment (**Supplementary Figure 4C**). This may be primarily attributed to differences in temperature transfer dynamics. The water bath provided rapid, near-instantaneous heat transfer due to direct contact with the pre-heated medium. In contrast, the heat plate required approximately 25 minutes to reach 36°C (**Supplementary Figure 3A**), resulting in a gradual temperature increase and a corresponding delay in fluorescence signal intensity.

We also performed time-lapse imaging to monitor the dynamics of YFP-HSP70 expression in hypocotyl cells during heat treatment and the subsequent recovery phase (**Figure 2C**). Notably, the augmentation of YFP-HSP70 intensity was predominantly observed in the nuclei, with only marginal increases in the cytoplasm of hypocotyl cells during the heat treatment (**Figure 2C** and **Supplementary Video 2**). To evaluate the heat plate’s suitability for recovery experiments, we switched off the heat plate after a 75-minute heat treatment and monitored changes during a 75-minute recovery phase. Interestingly, instead of a gradual decline, YFP-HSP70 fluorescence intensity showed a slight increase during recovery (**Figure 2D**). Additionally, small cytoplasmic speckles emerged in some seedlings during this phase, though no significant shift in the nucleocytoplasmic distribution of the YFP-HSP70 signal was observed (**Figure 2C** and **Supplementary Video 2**). It is difficult to determine whether the current heat plate setup is suitable for recovery experiments, as the observed increase in fluorescence intensity during the recovery phase may have been influenced by the residual warmth from the heat plate after it was switched off. The temperature decreases during recovery relied on environmental factors, as the system lacked a cooling mechanism.

### Design of a wireless mini-heater for live cell imaging

While the aluminum heat plate excelled in providing stable temperature conditions for live cell imaging, it was not without its limitations. Two significant challenges included its slow temperature rise during the oscillation phase and the restricted maximum temperature achievable at the specimen site (Point II). These issues were primarily attributed to the extended distance between the heater and the specimen, coupled with airflow at Point II.

**Figure 3:**
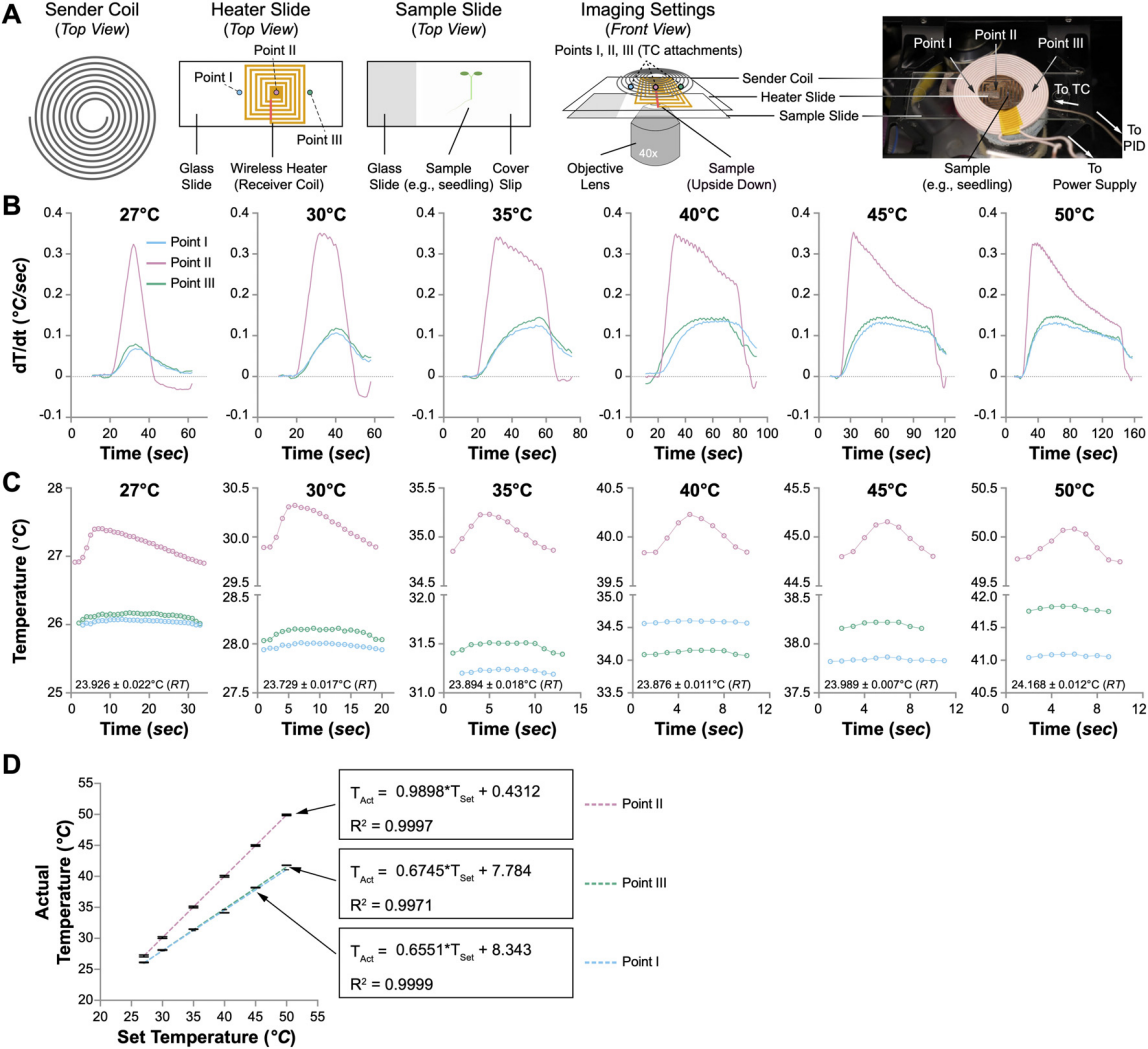
Performance of the wireless heater. **(A)** Illustration of the wireless heater settings for microscopical usage. The sample slide is upside down, with the coverslip facing the objective lens. The temperature changes at three spots (Points I, II, and III) were monitored with thermocouples (TCs). **(B)** The speed of temperature change (dT/dt, °C/sec) at Points I, II, and III when the wireless heater was set to different temperatures (27°C, 30°C, 35°C, 40°C, 45°C, and 50°C). **(C)** Temperature fluctuations at Points I, II, and III when the wireless heater was set to different temperatures. One oscillation cycle of the temperature changes is shown. The room temperature (RT) is indicated in each plot. **(D)** The correlation between the actual and set temperatures at Points I, II, and III. Linear regression is performed to evaluate the relationship between the set temperature (T_Set_) and the actual temperature (T_Act_). The formulas and R^2^ values are shown.

To address the limitations of the aluminum heat plate, we developed a compact heating device designed to be close to the specimen. While thermal fluid systems provide precise temperature control, their complexity and high cost are significant drawbacks. As a more straightforward, cost-effective alternative, we utilized copper foil tape shaped into a spiral coil to serve as the heating element. To optimize heating performance, the coil was designed with a 1 mm diameter and minimal spacing between adjacent coils. Its dimensions, 15 mm *×* 15 mm, ensure a fit on a standard glass slide. The coils were mounted on a glass slide (**Figure 3A**) to construct the wireless heater, which was then attached directly to the sample slide, aligning the copper receiver coil with the specimen’s center (**Figure 3A**). The copper receiver coil was heated via an alternating electromagnetic field generated by a sender coil placed atop it. This sender coil was connected to a PID controller, with a thermocouple affixed to the center of the receiver coil for precise thermal management. The minimal distance between the copper receiver coil and the specimen, coupled with direct temperature feedback, was designed to enable rapid and accurate temperature adjustments at the specimen site.

### The heating performance of the wireless mini-heater

We employed the same experimental setup previously utilized with the aluminum heat plate to ascertain the heating efficacy of the newly designed wireless mini-heater on live biological samples. Four-day-old Arabidopsis seedlings were mounted on a standard glass slide (75 mm *×* 25 mm), sandwiched between a coverslip (44 mm *×* 22 mm), and immersed in deionized water. Individual thermocouples were strategically placed at three points on the slide to capture and analyze thermal dynamics. These included Points I and III, located at the left and right edges of the receiver heater coil, and Point II, positioned precisely at the center of the wireless heater (**Figure 3A**). The system was tested under various temperature settings (27°C, 30°C, 35°C, 40°C, 45°C, and 50°C), allowing for comprehensive monitoring and documentation of temperature fluctuations and stability.

Initial assessments of the wireless heater’s thermal performance were carried out by monitoring temperature dynamics at the three points mentioned above during the initial heating stage, with the PID controller adjusted to various temperatures. We anticipated a significantly faster temperature rise at the specimen due to the proximity of the wireless heater, positioned directly above the Arabidopsis seedling. Confirming our hypothesis, the maximal rate of temperature increase at Point II exceeded 0.3°C/sec across all temperature settings, a rate more than 30 times greater than that observed at Point II of the aluminum heat plate (**Figure 3B**). This accelerated heating capability reduced the time required to reach the set temperatures at Point II, from less than 20 seconds at 27°C to just 2 minutes at 50°C, a duration at least 10 times shorter than that recorded with the aluminum heat plate (**Supplementary Figures 5A** and **3A**). However, the temperature elevation rate significantly decreased at the edges of the heater (Points I and III), achieving only one-third of the rate at Point II (**Figure 3B**). Moreover, although the temperature at Point II rapidly met the set values, the temperatures at Points I and III remained consistently lower, with variances ranging from less than 3°C at a PID setting of 27°C to nearly 10°C at 50°C (**Supplementary Figure 5A**).

Upon achieving the set temperatures at Point II, we monitored temperature oscillations at all three points, which were present regardless of the PID controller’s settings (**Supplementary Figure 5B**). Subsequently, we evaluated the temperature stability during the oscillation phase at various predefined settings. The oscillation periods at the three points remained consistent across all temperatures (**Figure 3C** and **Supplementary Figure 5C**). As the set temperature increased, the duration of these oscillations shortened, with average cycles lasting approximately 30 seconds at 27°C and reducing to under 9 seconds at 50°C (**Supplementary Figure 5C**). During these oscillation cycles, the temperature variance between the maximum and minimum values at Point II was maintained below 0.4°C, whereas for Points I and III, it was less than 0.15°C (**Supplementary Figure 5D**). In the oscillation phase, the temperature at Point II closely adhered to the set values (**Figure 3C**). In contrast, the temperatures at Points I and III consistently trailed, with more significant discrepancies at higher temperatures. Further analysis involved plotting the average temperatures recorded by the thermocouples at each point against the PID settings, revealing perfect linear correlations (**Figure 3D**). Linear regression of these data yielded exceptionally high R^2^ values of 0.9999, 0.9997, and 0.9971 for Points I, II, and III, respectively, underscoring the accuracy of our model. Notably, the slope at Point II was 0.9898, affirming the heater’s efficiency directly at the specimen site (**Figure 3D**).

### Testing the wireless mini-heater for live cell imaging of whole-mount seedlings

The observation that the wireless heater could heat and maintain a specimen at 50°C indicated its suitability for cell biology studies under heat stress and heat shock conditions. To evaluate this, we took advantage of a published Arabidopsis transgenic line of *eEF1Bβ1* (AT1G30230), a translation factor that is rapidly recruited to stress granule-like cytoplasmic foci upon high-temperature stress (Lohmann et al., 2023). We first tested this transgenic line, namely *p35S::YFP-eEF1Bβ1* (*YFP-eEF1Bβ1* for short), using a water bath set to 42°C. Five-day-old seedlings of *YFP-eEF1Bβ1* were subjected to this regimen for up to 120 minutes, with specimens collected every 30 minutes for immediate confocal microscopy. Images were captured from the epidermal cells of the cotyledons and the hypocotyl cells (**Supplementary Figure 4D**). Fluorescent signal was observed only in the cytoplasm in both guard cells and pavement cells in cotyledons and hypocotyl cells, consistent with the previous report and the function of eEF1Bβ1 in translational control. Without heat treatment, the YFP-eEF1Bβ1 protein was ubiquitously expressed and evenly distributed in the cytoplasm (0 min in **Supplementary Figure 4D**). Upon water bath heating, discrete cytoplasmic foci were observed after 30 minutes, and the number of cytoplasmic foci peaked after 60 minutes of heat treatment at 42°C. *YFP-eEF1Bβ1* seedlings incubated beyond 60 minutes seemed to accumulate fewer cytoplasmic foci. Given these observations, we elected to further study the dynamics of eEF1Bβ1-containing stress granule formation in this transgenic line using the wireless heater.

**Figure 4:**
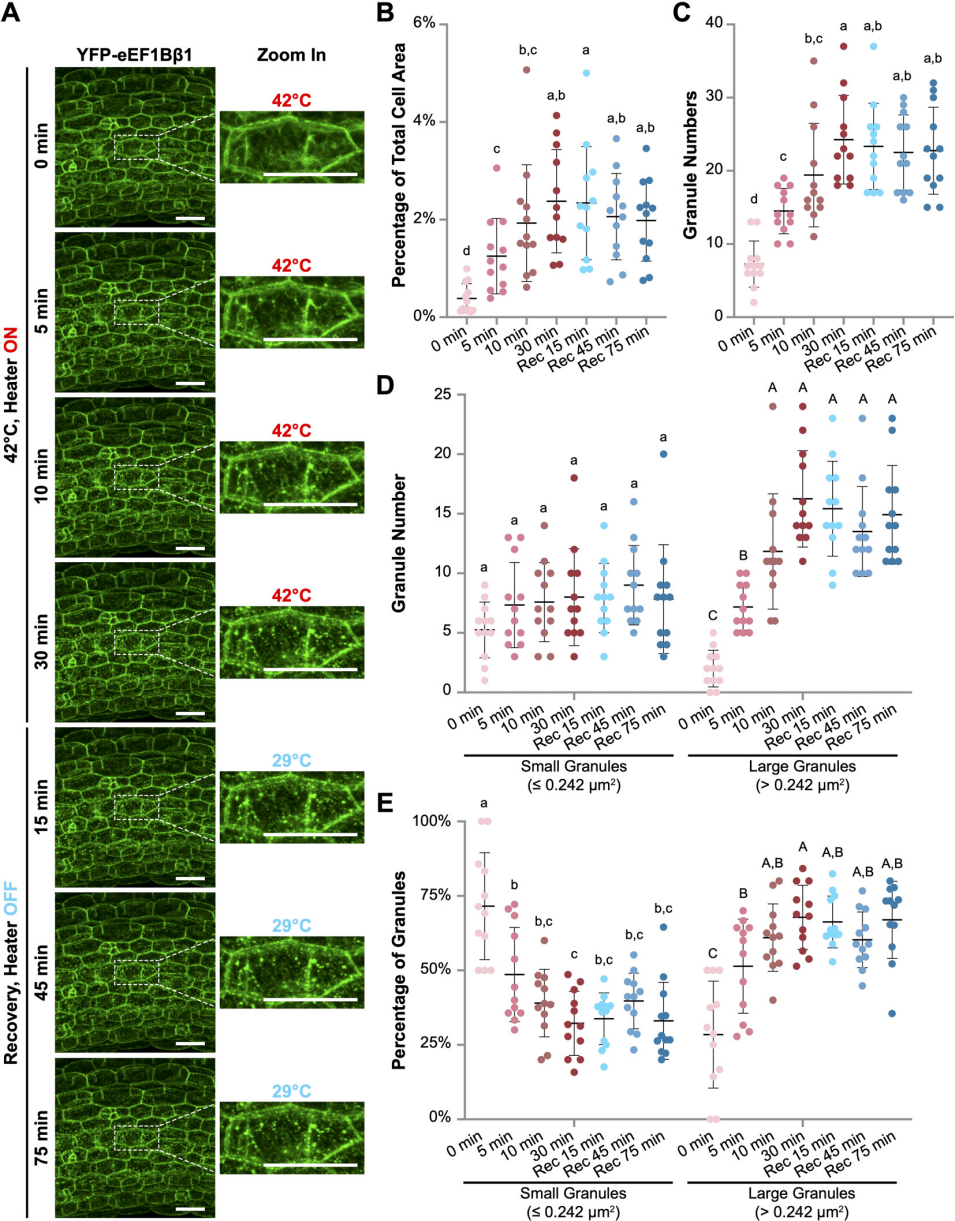
Heat shock induces the formation of YFP-eEF1Bβ1-containing cytoplasmic foci in Arabidopsis hypocotyl cells. **(A)** Confocal microscopy images of hypocotyl cells from Arabidopsis *35Spro::YFP-eEF1Bβ1* transgenic seedlings. Six-day-old seedlings were subjected to heat treatment at 42°C for up to 30 minutes using a wireless heater, followed by a recovery phase of up to 75 minutes. Enlarged views of a representative cell demonstrate the progressive formation of cytoplasmic foci during heat exposure. The sample temperature at each time point is indicated. Scale bar, 40 µm. **(B-E)** Quantitative analysis of granule size and number using ImageJ. **(B)** Granule area as a percentage of total cell area. **(C)** Total number of granules per cell. **(D)** Number of small granules (≤ 0.242 µm^2^) and large granules (> 0.242 µm^2^) per cell. **(E)** Percentage of small and large granules across different time points. Red dots represent data points from cells during heat treatment, while blue dots represent those from cells in the recovery phase. Different letters indicate statistically significant differences between time points (one-way ANOVA, n = 12, *p* < 0.05). Small and large granules are compared separately in D and E, lower- and upper-case letters being used, respectively.

We began by setting the wireless heater to 42°C and applying it directly to *YFP-eEF1Bβ1* seedlings mounted inside an adhesive imaging chamber on a microscope slide. The adhesive chamber minimized water loss, allowing for continuous imaging during heat treatment for more than 90 minutes. Prior to heating (0 min), no distinct granules were visible in most hypocotyl cells (**Figure 4A** and **Supplementary Video 3**). However, within 5 minutes of heating, discrete speckles started to form. Over the following 25 minutes, both the number and total area of YFP-eEF1Bβ1 granules increased significantly, peaking at 30 minutes (**Figures 4B, C**). Larger granules (> 0.242 µm^2^) progressively appeared, with both their size and number increasing during the heating phase (**Figures 4D, E**). To investigate recovery, we turned off the wireless heater after 30 minutes of heating and continued monitoring the dynamics for more than 75 minutes. Interestingly, despite the sample temperature decreasing to 30°C within 10 minutes of turning off the heater and remaining below 30°C thereafter, the number and size of YFP-eEF1Bβ1 granules did not change during the recovery phase (**Figures 4B-E**). We reasoned that the granules persisted due to the prolonged exposure to low heat (29°C), potentially delaying the recovery process. To enable more precise temperature control for both heat stress and recovery studies, we may need to integrate a cooling mechanism with the wireless heater in the future.

### Testing the wireless mini-heater for live cell imaging of Arabidopsis protoplasts

Having demonstrated the successful application of the wireless mini-heater for live cell imaging of whole-mount seedlings on microscope slides, we next explored its compatibility with other experimental setups, such as Petri dishes. Initially, we attempted to attach the wireless mini-heater to the bottom of standard 50-mm Petri dishes. However, when the PID was set to 42°C, the temperature reached only 28.5°C in an empty Petri dish and 32.5°C with a solution (data not shown). We reasoned that this issue was primarily due to the gap between the sender coil and receiver coil in standard Petri dishes, which significantly weakened the magnetic field and reduced heating efficiency. A more powerful sender coil or a thinner Petri dish might help address this limitation. To expand the applicability of the wireless heater to protoplasts and isolated plant cells (e.g., tobacco BY-2 cells), we focused on using chambered cover glass on microscope slides for the protoplast experiments. Since the wireless heater’s efficacy on microscope slides with adhesive chambers had been established with whole-mount seedlings, we hypothesized that it would also work effectively for protoplasts and isolated plant cells.

**Figure 5:**
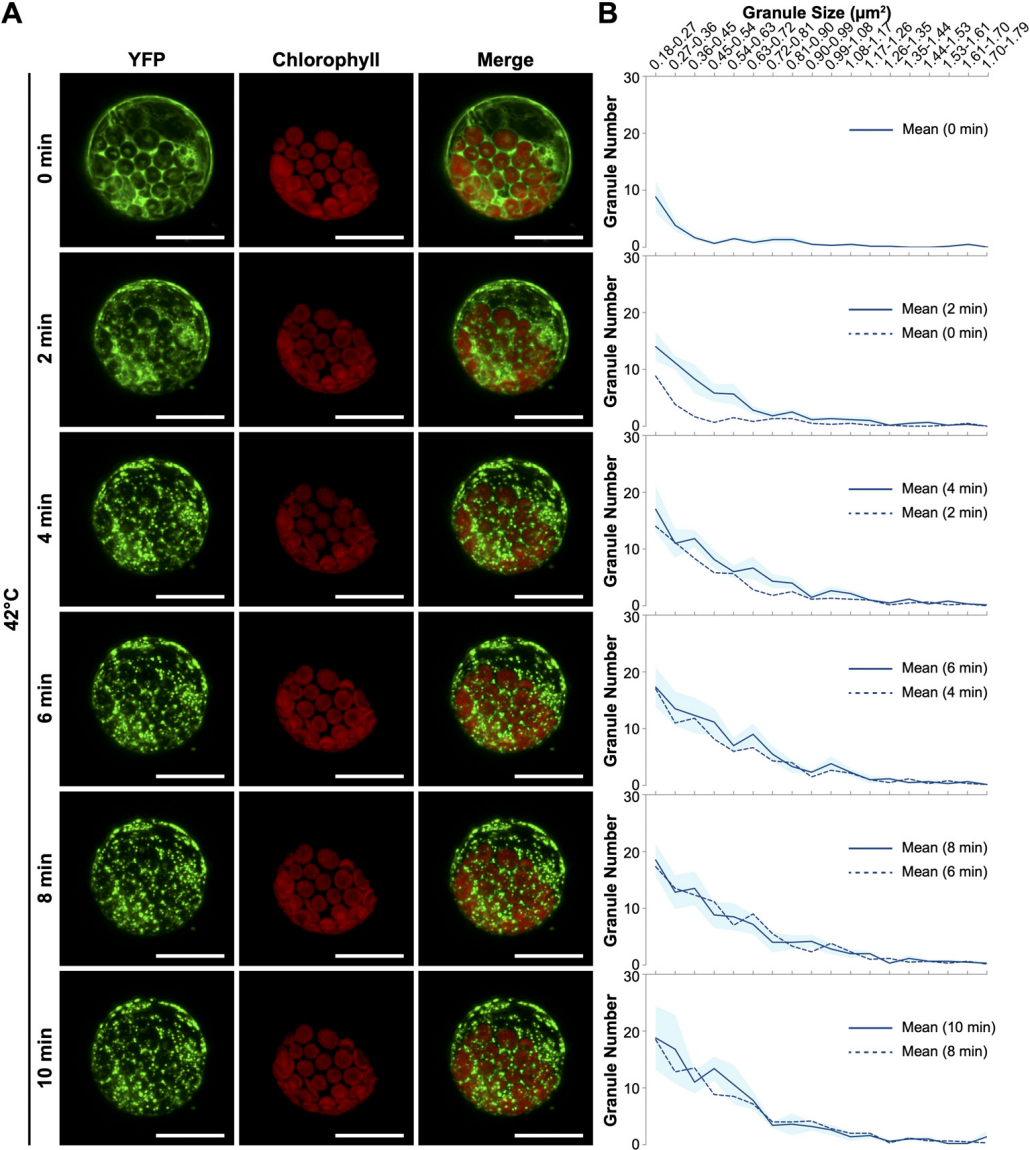
Rapid formation of eEF1Bβ1-containing cytoplasmic foci in response to heat shock. **(A)** Representative confocal images of protoplasts derived from the *35Spro::YFP-eEF1Bβ1* line. Protoplasts were subjected to heat treatment at 42°C for up to 10 minutes using a wireless heater. Images depict the formation of cytoplasmic foci at 0, 2, 4, 6, 8, and 10 minutes of heat exposure. Scale bar, 20 μm. **(B)** Quantitative analysis of granule size and number in *35Spro::YFP-eEF1Bβ1* protoplasts heated at 42°C. Cytoplasmic foci were quantified using ImageJ, and their distribution across 18 size bins (0.18 to 1.79 μm^2^) is presented for each time point. The dark blue line represents the mean number of granules within each bin, while the light blue shading indicates the standard error of the mean (SEM). The dashed line shows the mean number of granules at the previous time point for comparative reference. Data are based on the analysis of 6 protoplasts from two independent experiments.

To enhance the accuracy of quantifying YFP-eEF1Bβ1-containing granules, we investigated the protein dynamics within protoplasts derived from the *YFP-eEF1Bβ1* line. These freshly isolated protoplasts were secured in the adhesive imaging chamber attached to glass slides and subjected to a 42°C environment using a wireless heater. Time-lapse confocal microscopy documented the protein distribution both before and throughout a 10-minute heating period (**Supplementary Video 4**). The YFP-eEF1Bβ1 fluorescence was initially dispersed across the cytoplasm (**Figure 5A**). Although variability in fluorescence intensity existed across different cytoplasmic regions, discrete granules were scarcely detected before heating (0 min). Notably, cytoplasmic foci began to form after just 2 minutes of exposure to 42°C (**Figure 5A**), with particle analysis revealing a marked increase in granules ranging from 0.18 to 0.63 µm^2^ compared with pretreatment (**Figure 5B**). By the 4-minute mark, most of the YFP-eEF1Bβ1 signal was concentrated in distinct cytoplasmic foci, with an ongoing rise in the number of granules within the 0.36 to 0.90 µm^2^ size range. From 6 minutes onward, both the quantity and size of the YFP-eEF1Bβ1-containing foci stabilized, showing minimal changes (**Figure 5B** and **Supplementary Figure 6**).

## Discussion

### Custom heat plate enhances live cell imaging at moderately increased temperatures

While various temperature control systems are extensively applied in microscopic studies across different fields, their use in exploring thermal responses in plant cells remains notably rare. Among the few examples, Hamada et al. stand out for their innovative use of a heat incubator integrated directly with a microscope stage (Hamada et al., 2018). This setup allowed for the precise control of environmental temperature during live-cell imaging of Arabidopsis seedlings, revealing that stress granule formation is triggered above a critical temperature threshold of 34°C.

In this study, we introduced two heating devices designed to enhance live cell imaging for plant thermal biology studies in confocal microscopy. The first device, an aluminum heat plate, integrates seamlessly with the 160 mm × 110 mm sample holder compatible with mainstream microscopes, such as the Leica SP8 confocal microscope. This integration simplifies setup and offers versatility for accommodating various sample sizes, with a central aperture ensuring clear imaging paths (**Supplementary Figure 1**).

The heat plate effectively controls temperature increases up to 36°C on microscope slides (**Figure 1D**), which is ideal for moderate heat stress (30-36°C) and non-stressful high temperature (25-29°C) studies in many plant species, including the model dicotyledonous *Arabidopsis thaliana*. Future improvements could include using a more powerful power supply to enhance heating capacity and achieve temperatures suitable for heat shock studies (>37°C). We also observed uneven temperature distribution across the surface and the sample holder (**Supplementary Figure 2** and **Figure 1**). Although the current design features a temperature sensor placed directly opposite the heater (**Supplementary Figure 1**), this arrangement did not fully resolve the issue. Employing dual heaters on diagonal corners of the plate may improve temperature uniformity. Given the limited heating capacity of the plate and slight but observable temperature oscillations on the sample slide, we also encourage future users to determine the correlation between the PID set temperature and the actual sample temperature prior to live-cell imaging to ensure accurate heating.

Looking ahead, potential enhancements for the heat plate could significantly boost its utility. Integrating gas flow channels could benefit studies involving mammalian cells (**Supplementary Figure 1**), where controlling CO_2_ levels is crucial for maintaining cell health and behavior during imaging. Additionally, incorporating a thermoelectric cooler would allow for rapid and precise temperature adjustments (**Supplementary Figure 1**), facilitating both heating and cooling. This would not only extend the plate’s temperature range but also enhance its precision, which is vital for experiments requiring fast and strict thermal regulation. These improvements, while not tested in the current study, could open new avenues for the heat plate’s application in dynamic biological processes, such as heat shock responses and other temperature-dependent studies, ensuring more robust and versatile performance in sensitive and critical experiments.

### Wireless mini-heater provides rapid and precise thermal control for live cell imaging across a broad temperature range

The wireless mini-heater developed in this study significantly improves the precision of temperature control in plant heat stress research. It addresses the slow temperature rise and limited maximum temperature observed with the aluminum heat plate by providing rapid and efficient heating directly at the sample slide (**Figure 3**).

The operational principle of the wireless mini-heater relies on electromagnetic induction, similar to that used in wireless charging for cell phones. When an alternating current (AC) flows through the sending coil, it generates a fluctuating magnetic field. According to Faraday’s law of electromagnetic induction, this changing magnetic field induces an electromotive force (EMF) in a nearby conductive coil. When the receiver coil, positioned within this magnetic field, experiences induced EMF, it generates an electric current. As per Joule’s Law, the flow of this current through the resistive material of the coil generates heat. This process effectively increases the surface temperature of the receiving coil, enabling it to function as a heater.

Our wireless mini-heater, which incorporates a copper foil tape shaped into a spiral receiver coil that directly contacts the sample (**Figure 3A**), offers superior heat transfer efficiency and reduces thermal lag compared to traditional heat stages and chambers. This close proximity to the specimen enhances response time, achieving temperature increases more than 30 times faster than the aluminum heat plate (**Figure 3B**). Such rapid heating is crucial for accurately replicating the swift temperature changes characteristic of natural heat stress conditions. Additionally, the wireless mini-heater provides a more uniform temperature distribution near the thermocouple location, with testing showing that the central point (Point II) maintains temperatures close to the set values with minimal fluctuations (**Figure 3D**). This stability is vital for live cell imaging, where even minor temperature variations can significantly impact cellular behavior.

The heater’s effectiveness was demonstrated through experiments with Arabidopsis seedlings, successfully maintaining temperatures up to 50°C (**Figure 3B**), which is suitable for severe heat stress studies across various plant species beyond Arabidopsis. The device also proved instrumental in observing stress granule dynamics in both whole-mount seedlings and protoplasts (**Figures 4** and **5**). For example, when Arabidopsis protoplasts were subjected to 42°C using the wireless mini-heater, YFP-eEF1Bβ1-containing cytoplasmic foci appeared within just 2 minutes, consistent with similar observations in *eIF4A2-GFP* plants (Hamada et al., 2018).

### New heating devices for live cell imaging are highly affordable without compromising their functionality

In the context of live cell imaging, where budget constraints often limit access to high-cost equipment, our heating devices stand out for their performance and cost-effectiveness. Both the aluminum heat plate and the wireless mini-heater are designed to be economically feasible, making them ideal for small laboratories with limited funding. Commercially available heating devices can be prohibitively expensive, with prices ranging from $2,000 to over $30,000 for advanced systems, making them inaccessible to many research groups (Rossi et al., 2022; O’Carroll et al., 2023; Worcester et al., 2023). In contrast, our devices offer a more accessible solution, with costs significantly lower than these commercial options.

A detailed breakdown of costs associated with our devices (**Supplementary Tables S1** and **S2**) showcases their economic viability. The total cost for constructing the aluminum heat plate is approximately $160, while the wireless mini-heater costs about $300. These costs are comparable to other custom-built solutions reported in the literature, where prices range from $270 to $350 (Rossi et al., 2022; O’Carroll et al., 2023; Worcester et al., 2023), demonstrating that our heating devices are competitively priced. Additionally, the cost of the wireless mini-heater could be further reduced by substituting the current multi-functional PID controller, which costs over $200, with a more affordable standard PID controller priced at around $30.

The economic efficiency of our designs does not detract from their functionality. Our devices, especially the wireless mini-heater, not only match but sometimes surpass the capabilities of more expensive systems in the market, particularly in rapid and precise temperature control (**Figure 3** and **Supplementary Figure 5**). The simplicity of their design and the straightforward fabrication process make these devices exceptionally user-friendly, requiring minimal technical skills in electronics or mechanical engineering. This ease of assembly and operation ensures that our heating devices are accessible to researchers from a broad range of scientific backgrounds, facilitating advanced studies in thermal biology without excessive expenditure.

### Further design enhancements will improve the usability and performance of the new heating devices for live cell imaging

Heat uniformity and focus drift present significant challenges in both designs. The aluminum heat plate exhibited uneven temperature distribution across its surface and sample holding region (**Figure 1** and **Supplementary Figure 2**), while the wireless heater, despite improving heat distribution, still faced issues with temperature uniformity, particularly across the sample slide (**Figure 3**). The temperature at the central point of the wireless heater (Point II) was closer to the set temperature, but temperatures at Points I and III, located ~7 mm away, were consistently lower—ranging from ~1°C at 27°C to nearly 9°C at 50°C (**Supplementary Figure 5B**). The temperature variation on the wireless heater is possibly caused by uneven magnetic field distribution and localized eddy currents. The magnetic field generated by the sender coil is not uniform across the receiving coil due to differences in position and coil geometry. Stronger magnetic fields tend to occur near the center of the coil, while weaker fields are found towards the edges. This uneven field induces localized eddy currents, which are circular currents within the copper wire. These eddy currents generate heat, with higher heating in regions where the eddy currents are concentrated. Additionally, the fine copper wire of the coil introduces thermal resistance, meaning heat cannot easily distribute evenly across the wire. As a result, areas with stronger eddy currents experience higher temperatures, while regions with weaker currents stay cooler, leading to an overall temperature imbalance. Improving temperature uniformity could involve using a larger sending coil to obtain a more evenly distributed magnetic field. Additionally, applying a thin film of Indium tin oxide (ITO), known for its electrical conductivity and optical transparency, to the sample slide or the receiving coil slide could further improve temperature distribution, benefiting both the wireless heater and the aluminum heat plate. Another promising approach is to integrate a small incubator with controlled humidity and temperature, providing a more stable environment for long-term experiments, which would help mitigate temperature variations and improve overall heat uniformity. However, it’s important to note that these enhancements would likely lead to increased costs, potentially making the devices less accessible to budget-conscious laboratories.

Another challenge faced by both devices was the inability to maintain the required temperature during the recovery phase due to the lack of a cooling mechanism. In the heat plate experiment, after heat stress was terminated, the intensity of YFP-HSP70 fluorescence in hypocotyl cells continued to rise slightly, indicating a delay in the recovery process (**Figures 2C, D**). This occurred even as the temperature at the sample site decreased to around 30°C after the heat plate was turned off. Similarly, in the wireless mini-heater experiment, the number and size of YFP-eEF1Bβ1 granules remained unchanged during the 75-minute recovery phase after the heater was switched off, despite the temperature dropping to below 30°C (**Figure 4**). These observations suggest that the temperature remained elevated enough to prevent the expected recovery response. To address this issue, future designs should incorporate a cooling mechanism that enables more precise control of both heat stress and recovery temperatures.

Focus drift has been a persistent challenge in live cell imaging, particularly when conducted at high magnifications (Silfies et al., 2013). One of the primary contributors to focus drift is evaporation, which becomes especially problematic during long-term imaging experiments. Evaporation not only leads to a gradual loss of focus as the sample dries out but also exacerbates thermal drift by altering the local humidity and temperature. To address evaporation, our use of adhesive imaging chambers has proven beneficial (**Supplementary Video 3** & **4**); however, for longer experiments exceeding one hour, a more closed system with humidity control could be implemented to prevent focus drift effectively.

Thermal drift remains another significant factor, where temperature fluctuations—caused by air conditioning, central heating units, intense microscope illumination, or uneven heating of objectives and stages—lead to shifts in the focal plane (Silfies et al., 2013). In our designs, thermal drift is further aggravated by the differential expansion and contraction rates of materials in the heating devices and microscope components. Integrating an objective heater with our heating systems could help maintain a consistent temperature across the microscope’s optical path, thereby reducing focus shifts during extended imaging sessions.

Coverslip flex, which results from thermal gradients, also contributes to focus drift (Silfies et al., 2013). Although the use of adhesive coverslips in our current designs may help reduce flex, further enhancements are needed. Improving temperature control within the sample slide, as previously discussed, could stabilize the coverslip and help maintain focus. Additionally, applying a conductive, transparent ITO coating on the coverslip could offer a solution by maintaining uniform temperature, though it’s essential to manage any contractions or expansions that may temporarily disrupt focus. While these modifications could increase the system’s complexity, they hold the potential to significantly enhance the reliability of focus during long-term live cell imaging.

In summary, the wireless mini-heater, along with the aluminum heat plate, represents a significant advancement in temperature control for live cell imaging, particularly in plant biology research under various thermal stresses. Both devices offer straightforward designs and ease of fabrication, making them accessible alternatives to more traditional, often cumbersome heating systems used in confocal microscopy setups. The wireless mini-heater, with its rapid heating response and uniform temperature distribution, excels in providing precise thermal control essential for studying dynamic cellular processes. Meanwhile, the aluminum heat plate offers moderate heating suitable for a range of experimental conditions, with potential future enhancements to improve temperature uniformity and range. Together, these devices not only enhance experimental flexibility but also ensure reliable outcomes, making them valuable tools for researchers working in environments with limited resources. Future iterations could incorporate additional features, such as improved thermal feedback mechanisms and integration with objective heaters, a cooling component, humidity control incubators, and ITO-coated coverslips, to further expand their capabilities and application scope.

## Methods

### Design and fabrication of the aluminum heat plate

The heat plate insert was designed using Fusion 360 to align perfectly with the dimensions of the standard 160 mm × 110 mm sample holder compatible with mainstream microscopes, including the Leica SP8 confocal microscope. This advanced heat plate can accommodate standard slides and 35 mm culture dishes, offering precise control over temperature and gas mixtures. It was crafted in the Department of Physics and Astronomy machine shop at the University of Mississippi. The primary heat source, a 10 Watts/In^2^ self-adhesive polyimide flexible heater (Omega Engineering Inc., Norwalk, CT), is strategically embedded within the plate. An adjustable DC converter transformer (SHNITPWR, Hong Kong, China) was integrated to facilitate fine-tuned temperature adjustments, providing a versatile output range of 3V to 24V. A DC 12V 1-relay temperature controller (Bayite Technology Co., Shenzhen, China) was incorporated to ensure precise temperature management. This system features a highly sensitive NTC thermistor encapsulated in transparent epoxy resin (Janchun Technology Co., Shenzhen, China) linked to the temperature controller. The controller operates in heat mode, activating the relay whenever the temperature falls just 1 degree below the set point and deactivating it upon reaching the desired temperature. This configuration guarantees precise and consistent temperature management suitable for diverse scientific applications.

### Design and fabrication of the wireless heater

The wireless heater was constructed using copper foil tape (LOVIMAG, Nanjing Anshiqing E-commerce Co., Ltd, China), which is 1 inch wide and sourced from Amazon. The design of the heater pattern was created in Creo CAD Software and then transferred to a laser cutter for precise fabrication. Alternatively, the pattern can be manually cut, further reducing complexity and cost. Once cut, the pattern was affixed to a glass slide to assemble the heater. To ensure insulation without covering connectivity, magic tape (3M) was strategically placed between two critical points. An additional layer of copper tape was then applied over the magic tape to bridge these points, completing the circuit and enabling functionality.

### Construction of the closed-loop proportional-integral-derivative control heating system

The fabricated heater was placed directly above a 30W (12V with 2.5A) wireless charging module (the sender coil) from Taidacent (Shenzhen Taida Century Technology Co., Ltd., China). Adjacent to the heater, a 36-gauge K-type thermocouple (Omega Engineering Inc., Norwalk, CT) was installed to monitor the temperature accurately. This thermocouple was connected to an OMEGA CNPT-330 PID controller, which both senses the temperature and controls the heating mechanism. The wireless charging module, integral to the system’s operation, received power from a 12V supply managed by the PID controller. Users can directly set the desired target temperature on the PID controller. Before deployment, the system underwent a thorough characterization process to determine the optimal settings for the proportional, integral, and derivative parameters, ensuring the heating system operates within its most influential parameters.

### Plant materials and growth conditions

All the Arabidopsis mutants and transgenic lines used in this study are in the Col-0 background. The null *hsp70-4-2* mutant was obtained from the Arabidopsis Biological Resource Center (stock# SALK_088253C). The *35Spro::YFP-eEF1Bβ1* line was previously generated and described (Lohmann et al., 2023). The *AtHSP70-4pro::YFP-AtHSP70-4* transgenic line was generated in this study (see below). Homozygosity of the seeds was confirmed by plant selection markers before they were used for confocal microscopy. Seeds were surface sterilized consecutively with 70% ethanol for 1 minute and bleach (3% sodium hypochlorite) for 10 minutes before being plated on half-strength Murashige and Skoog (1/2 MS) media supplemented with Gamborg’s vitamins (MSP06, Caisson Laboratories, North Logan, UT), 0.5 mM MES (pH 5.7), and 0.8% (w/v) agar (A038, Caisson Laboratories, North Logan, UT). Seeds were stratified in the dark at 4°C for four days to synchronize germination and were grown under long-day conditions (16 h light/8 h darkness) with 125 µmol m^-2^ s^-1^ white light at 20°C in LED chambers (Percival Scientific, Perry, IA) unless otherwise stated. Fluence rates of light were measured using an Apogee PS200 spectroradiometer (Apogee Instruments Inc., Logan, UT), and the chamber temperature was calibrated with two digital thermometers.

### Plasmid construction and generation of transgenic lines

All PCR reactions were executed utilizing the Q5 High-Fidelity DNA Polymerase, and ligation reactions were carried out using the NEBuilder HiFi DNA Assembly Master Mix (New England Biolabs Inc., Ipswich, MA). In the pursuit of generating the binary vector to facilitate the construction of the *AtHSP70-4pro::YFP-AtHSP70-4* transgenic line, the entire coding sequence (CDS) of *AtHSP70* (AT3G12580) was fused with a sequence encompassing a *3×HA-tag*, a *YFP* moiety, and a *PT linker* (PTPTPTPTP) when being cloned into the binary vector pJHA212H at the EcoRI and BamHI restriction sites. Upstream the open reading frame, a 1,362 bp fragment of the *AtHSP70-4* promoter sequence was integrated to drive the expression of *3×HA-YFP-(PT)_4_P-AtHSP70-4*. The primer sequences employed for assembling this plasmid are presented in **Supplementary Table S3**.

To generate the *AtHSP70-4pro::YFP-AtHSP70-4* transgenic line, the null *hsp70-4-2* mutant (SALK_088253C) was transformed with the above-described pJHA212H/*AtHSP70-4pro::3×HA-YFP-(PT)_4_P-AtHSP70-4* plasmid using the *Agrobacterium*-mediated floral dip method. The resultant transformants were screened on 1/2 MS medium supplemented with 100 µg/ml hygromycin. Notably, a minimum of ten independent lines were chosen, which exhibited a Mendelian segregation ratio of approximately 3:1 for hygromycin resistance in the T2 generation. Transgene expression was evaluated by immunoblots using the anti-GFP antibody (Ab290, Abcam), and one line demonstrating intermediate expression levels (line #13) was chosen for the study. All experimental work was conducted using T3 self-progeny derived from homozygous T2 plants.

### Protoplast preparation

Leaf samples were harvested from 3- to 4-week-old soil-grown plants and sectioned into 1 mm strips using a sterile razor blade. These strips were then immersed in an enzymatic solution comprising 20 mM MES (pH 5.7), 0.4 M mannitol, 20 mM KCl, 1.5% cellulase R10, 0.4% macerozyme R10 (both from Yakult Pharmaceutical Industry Co., Ltd., Japan), 10 mM CaCl_2_, and 0.1% BSA. The mixture was incubated in complete darkness at 21°C with gentle agitation for 5-9 hours. Following incubation, protoplasts were collected by centrifugation at 200 g for 4°C with a gentle deceleration, and the supernatant was carefully discarded. The isolated protoplasts were resuspended in W5 medium, including 2 mM MES (pH 5.7), 154 mM NaCl, 125 mM CaCl_2_, 5 mM KCl, and 0.4 M mannitol. They were promptly used for subsequent heat treatment and confocal microscopy analysis.

### Heat treatment

In the water bath heat treatment, five-day-old seedlings grown on ½ MS media in 100 mm × 100 mm square Petri dishes sealed with Parafilm were subjected to heat in a water bath (VWR International, Radnor, PA). For immunoblots, seedlings underwent a 2-hour exposure at 36°C. For confocal imaging, the *AtHSP70-4pro::YFP-AtHSP70-4* line was observed after intervals of 0, 30, 60, 90, and 150 minutes at 36°C, and the *35Spro::YFP-eEF1Bβ1* line after 0, 30, 60, 90, and 120 minutes at 42°C.

In the heat plate treatment, seedlings were mounted on microscope slides (Fisher Scientific, Hampton, NH) in ddH_2_O, covered with a cover slip (Thermo Fisher Scientific, Waltham, MA), and placed on the heat plate before loading onto the Leica SP8 confocal microscope. The temperature was monitored using a thermocouple and a Teledyne FLIR E76 thermal camera (Teledyne FLIR, Wilsonville, OR).

For the wireless heater treatment, seedlings or isolated protoplasts were mounted on microscope slides in ddH_2_O or W5 medium and enclosed with a single-chamber adhesive coverslip, namely HybriWell Sealing System (Grace Bio-Labs, Bend, OR), to minimize water evaporation. This setup was positioned on the slide holder of the Leica SP8 confocal microscope, with the heating coil directly above, and temperatures were recorded using a Teledyne FLIR E76 thermal camera (Teledyne FLIR, Wilsonville, OR).

### Image acquisition

Confocal images were captured with a Leica SP8 Inverted Confocal Microscope (Leica Microsystems Inc., Buffalo Grove, IL), utilizing a 40× immersion objective lens. Fluorescent samples were illuminated using a White Light Laser (WLL) set to 20% power and an excitation wavelength of 500 nm. For visualizing YFP and chlorophyll fluorescence, emission wavelengths were set to 530-575 nm and 650-750 nm, respectively, with detection performed using a HyD detector. Image acquisition was conducted in a 1024×1024 pixel format, incorporating bidirectional scanning and averaging two- or four-line scans for enhanced clarity and resolution.

### Image analysis

For analyzing YFP-eEF1Bβ1-containing cytoplasmic foci, confocal images are processed using FIJI/ImageJ following a structured workflow designed to enhance clarity and ensure consistency. The process begins with despeckling, using the Despeckle filter (Process > Noise > Despeckle) to reduce image noise. This step is followed by unsharp masking (Process > Filters > Unsharp Mask), which sharpens the images and enhances particle visibility. Image normalization is then performed using the Normalize Local Contrast plugin (Plugins > Integral Image Filters > Normalize Local Contrast) to correct for variations in particle intensities. Additional image cleanup is carried out as necessary to refine particle separation. Thresholding is applied (Image > Adjust > Threshold) to differentiate particles from the background, and particle size and shape descriptors are analyzed using the Analyze Particles tool (Process > Analyze > Analyze Particles). This comprehensive workflow ensures that all images are processed under identical conditions to accurately analyze particle characteristics, with the procedures standardized via an attached macro. Following particle analysis in hypocotyl cells, cytoplasmic foci between 0.161 and 4.278 µm^2^ and circularity larger than 0.5 were selected for final analysis. Following particle analysis in protoplasts, cytoplasmic foci between 0.18 and 1.79 µm^2^ and circularity larger than 0.6 were selected for final analysis.

### Accession numbers

Accession numbers are as described by TAIR (https://www.arabidopsis.org) as follows: HEAT SHOCK 70 KDA PROTEIN 4 (HSP70-4), AT3G12580; EUKARYOTIC ELONGATION FACTOR 1B BETA 1 (eEF1Bβ1), AT1G30230.

## Data Availability

The datasets presented in this study can be found in online repositories. The names of the repository/repositories and accession number(s) can be found below: https://www.ncbi.nlm.nih.gov/genbank/, GSE196969.

## Supplementary material

Supplementary Video 1 Time-lapse video of YFP-AtHSP70-4 accumulation in Arabidopsis epidermis treated with the heat plate at 36°C.

Supplementary Video 2 Time-lapse video of YFP-AtHSP70-4 accumulation in Arabidopsis hypocotyl treated with the heat plate at 36°C.

Supplementary Video 3 Time-lapse video of YFP-eEF1Bβ1-containing cytoplasmic foci in Arabidopsis hypocotyl treated with the wireless heater at 42°C.

Supplementary Video 4 Time-lapse video of YFP-eEF1Bβ1-containing cytoplasmic foci in an Arabidopsis protoplast treated with the wireless heater at 42°C.
