# Supplementary material for: Developing affordable and efficient heating devices for enhanced live cell imaging in confocal microscopy
Source: Front Plant Sci. 2025 Jan 10;15:1499831. doi: 10.3389/fpls.2024.1499831 (PMC11760603; doi:10.3389/fpls.2024.1499831)
Supplement: Supplementary file 1 [file DataSheet1.pdf]

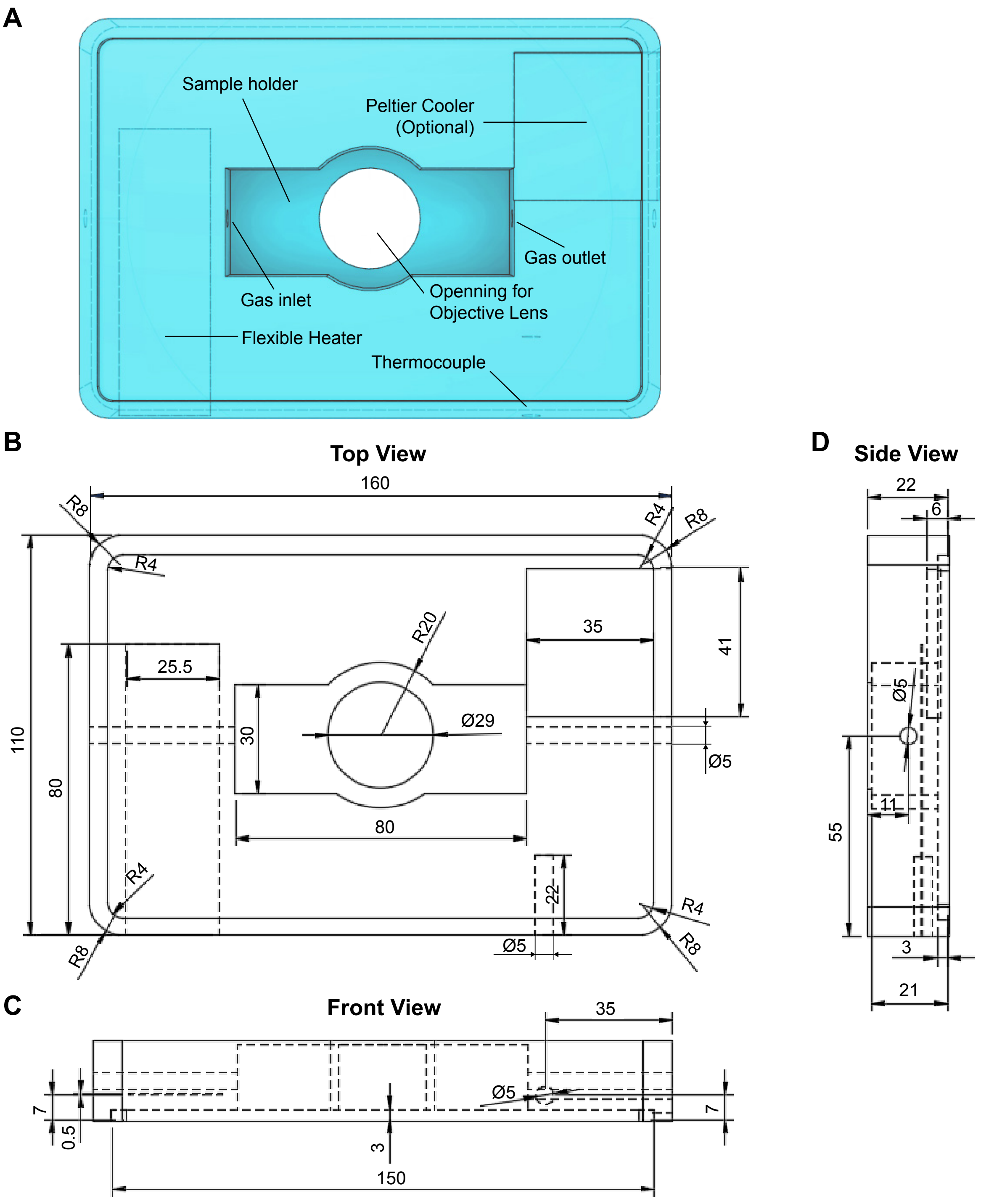


**Supplemental Figure 1. Design and dimensions of the heat plate.** (A) Top view of the heat plate with functional parts labeled. (B-D) Dimensions of the heat plate.

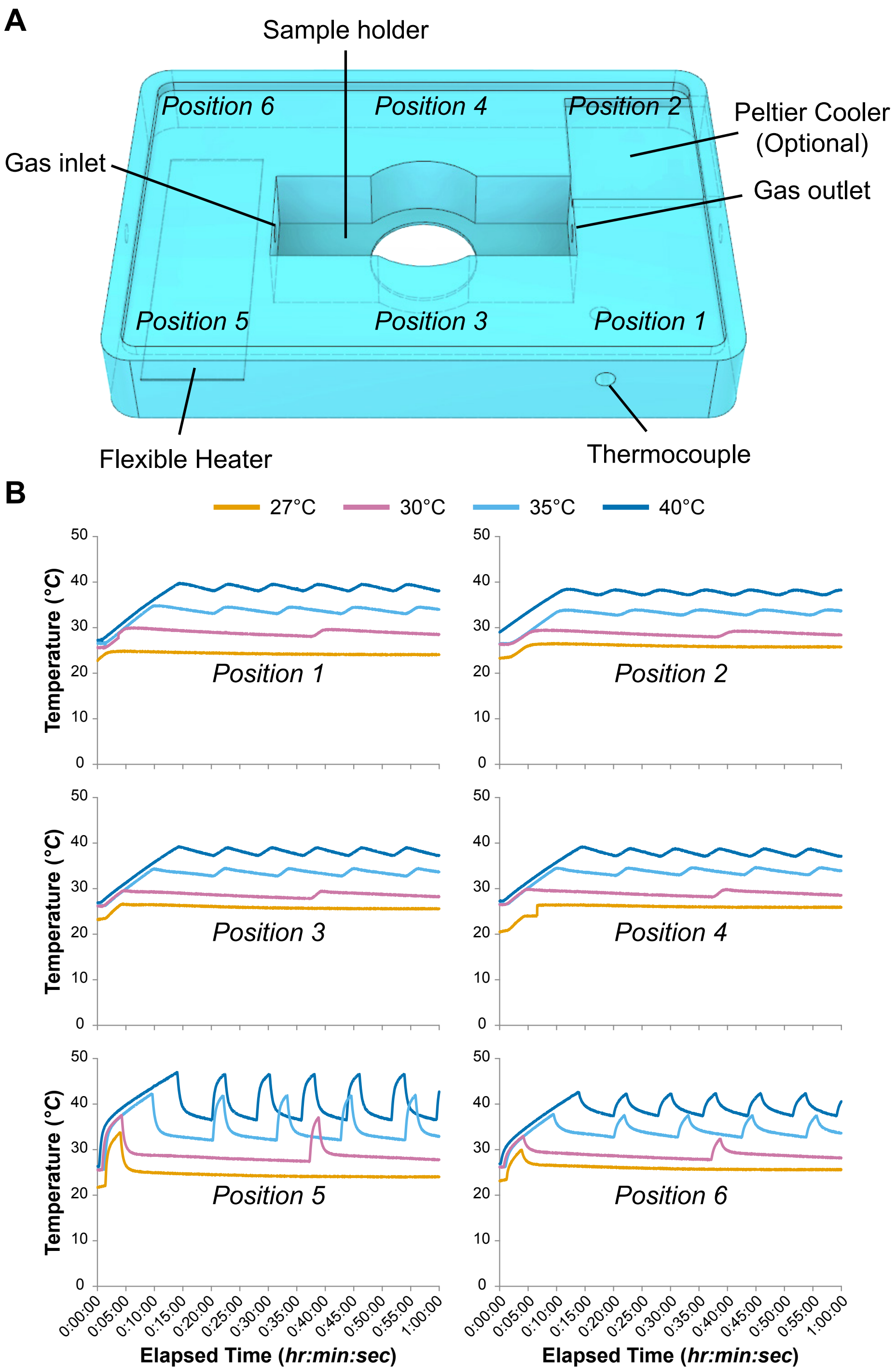


**Supplemental Figure 2. The spatial distribution of temperatures across distinct locations on the heat plate.** (A) The schematic depiction indicates the precise positions of six designated points on the heat plate for temperature monitoring, emphasizing the relative orientation of each position in relation to both the flexible heater and the thermocouple. (B) Temporal profiles depict the recorded temperature variations at these six specified positions when the heat plate was set to varying temperature settings (27°C, 30°C, 35°C, and 40°C). The presented data encompasses the initial hour of experimentation. It is noteworthy that the ambient room temperatures varied across experimental conditions, thereby influencing the disparate initial temperatures (00:00:00) at each setting.

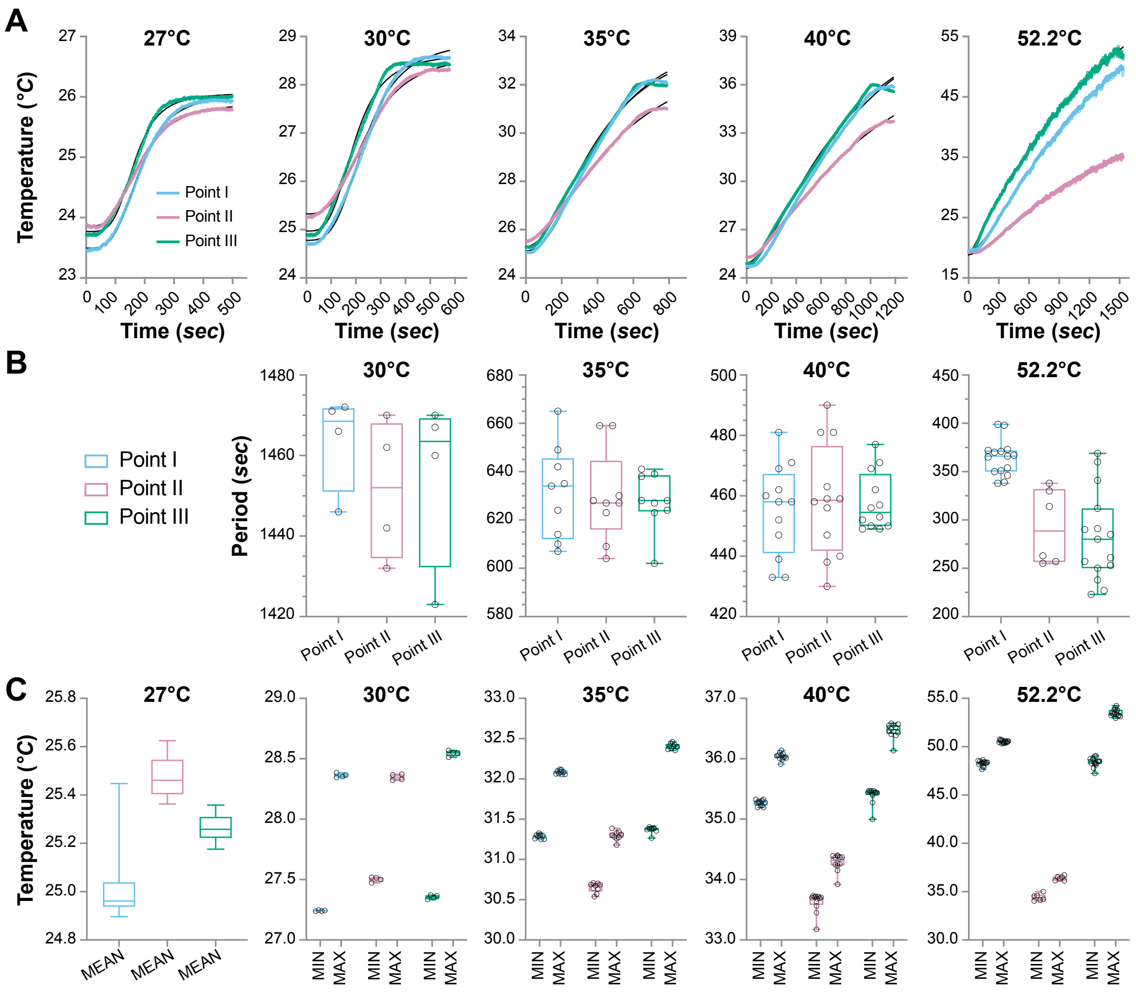


**Supplemental Figure 3. Performance of the heat plate.** (A) Temperature increases at Points I, II, and III when the heat plate was set to different temperatures (27°C, 30°C, 35°C, 40°C, and 52.2°C). Nonlinear regression (black sigmoidal curve) was performed for each plot. (B) The time period of the temperature oscillation at Points I, II, and III. (C) The average temperature (27°C) or the average minimal and maximal temperatures of each oscillation cycle (30°C, 35°C, 40°C, and 52.2°C) at Points I, II, and III.

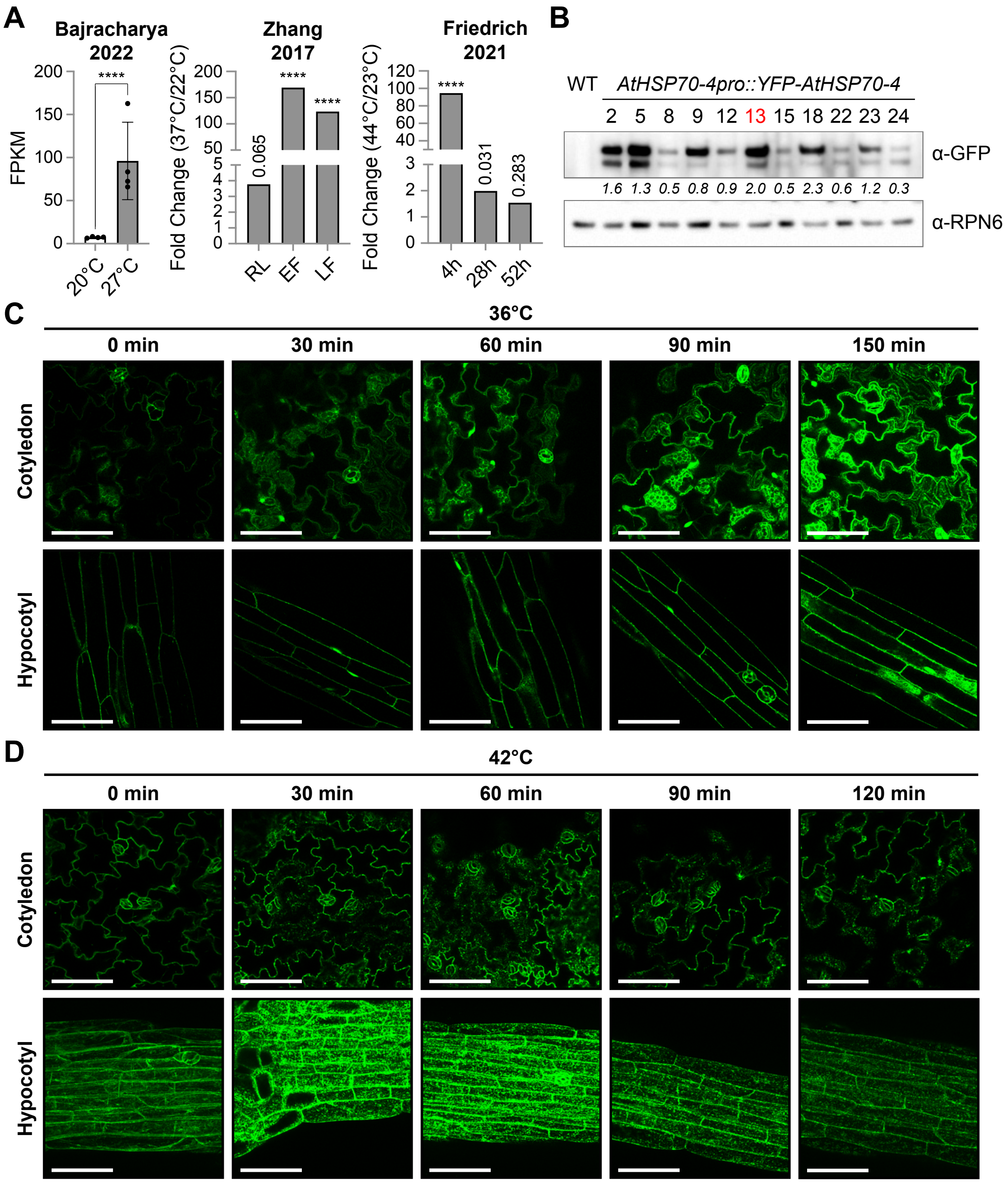


**Supplemental Figure 4. Performance of two marker lines in heat treatments.** (A) RNA-seq data from three studies indicate that *AtHSP70-4* can be highly induced by a wide range of high temperatures: 27°C by Bajracharya et al., 2022; 37°C by Zhang et al., 2017; 44°C by Friedrich et al., 2021. RL, rossette leaves; EF, early flowers; LF, late flowers. **** indicates a q-value lower than 0.0001. (B) Immunoblots of eleven individual single-insertion Arabidopsis lines expressing AtHSP70-4pro::YFP-AtHSP70-4. T3 homozygous seedlings in the *athsp70-4* mutant background (SALK_088253) were grown in a long-day condition (16 h day/8 h night, 30 µmol $m^{-2}$ $s^{-1}$ white light) for five days and heat treated at 36°C for 2 hours in a water bath. The expression of YFP-AtHSP70-4 was evaluated by an anti-GFP (Ab290) antibody. RPN6 was used as an internal control. Italicized numbers indicate the relative expression levels of YFP-AtHSP70-4 relative to those of RPN6. Line #13 was selected for imaging analyses. (C) Confocal images of *AtHSP70-4pro::YFP-AtHSP70-4* line #13 after heat treatment at 36°C for up to 150 min in a water bath. Images were taken from the epidermis of cotyledons and the middle section of hypocotyls in 5-day-old seedlings grown in a long-day condition (16 h day/8 h night, 30 µmol $m^{-2}$ $s^{-1}$ white light). Scale bar, 100 µm. (D) Confocal images of *35Spro::YFP-eEF1Bβ1* line in the *eef1b β1* mutant background (SALK_026418) after heat treatment at 42°C for up to 120 min in a water bath. Images were taken from the epidermis of cotyledons and hypocotyls in 5-day-old seedlings grown in a long-day condition (16 h day/8 h night, 30 µmol $m^{-2}$ $s^{-1}$ white light). Scale bar, 100 µm.

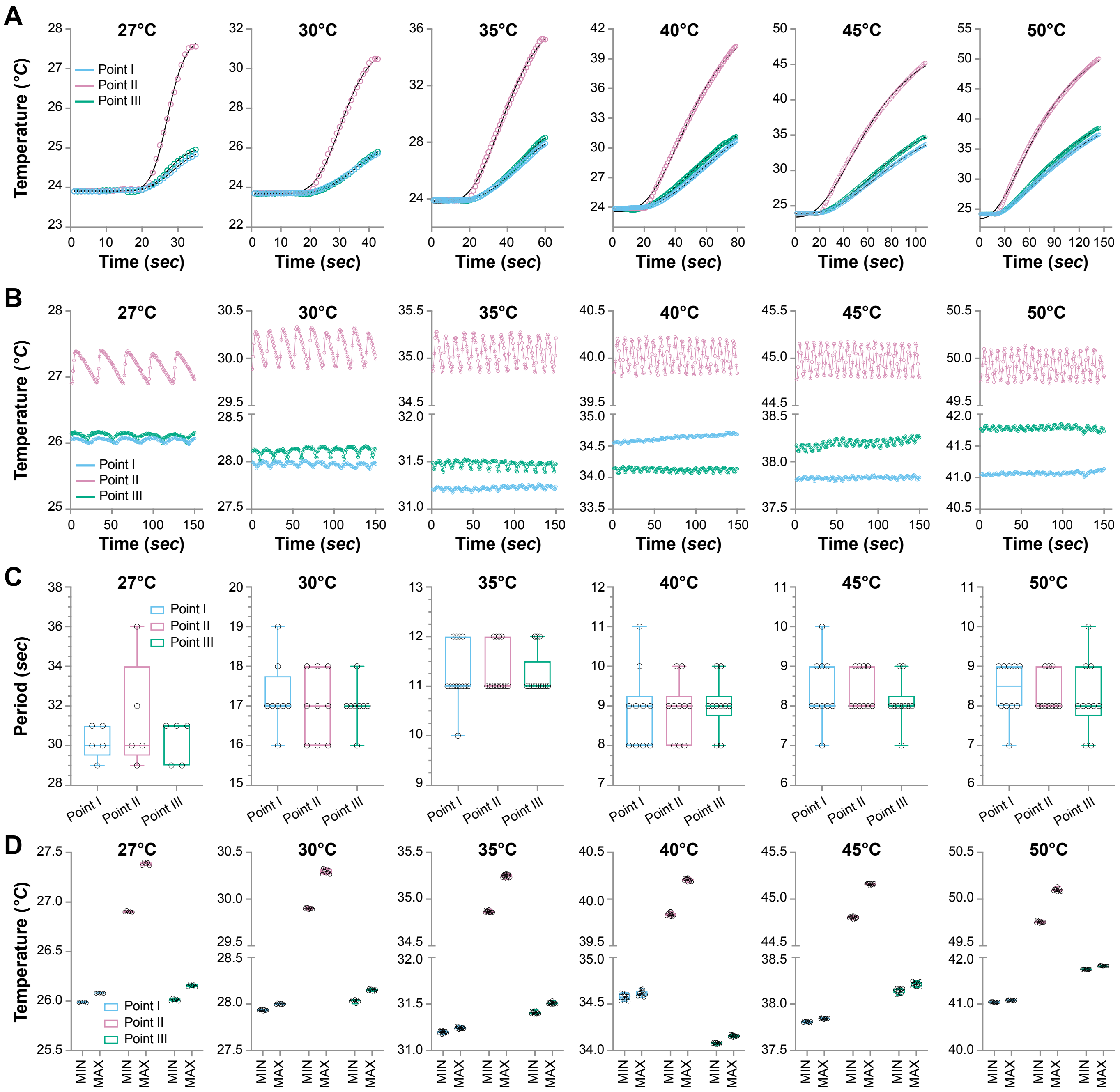


**Supplemental Figure 5. Performance of the wireless heater.** (A) Temperature increases at Points I, II, and III when the wireless heater was set to different temperatures (27°C, 30°C, 35°C, 40°C, 45°C, and 50°C). Nonlinear regression (black sigmoidal curve) was performed for each plot. (B) Temperature fluctuations within a 150-second time frame at Points I, II, and III after the temperature reached the set value. (C) The time period of the temperature oscillation at Points I, II, and III. (D) The average minimal and maximal temperatures of each oscillation cycle at Points I, II, and III.

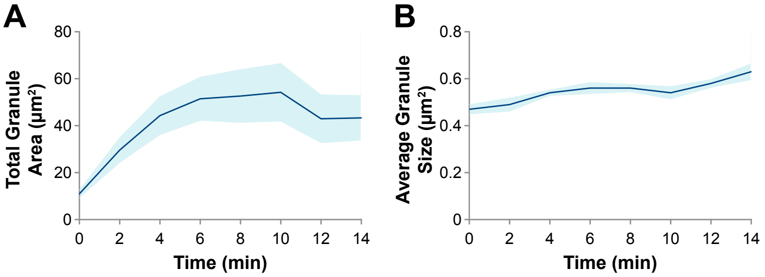


**Supplemental Figure 6. Characteristics of YFP-eEF1Bβ1-containing cytoplasmic foci in response to heat shock.** Quantitative analysis of total granule area (A) and average granule size (B) in *35Spro::YFP-eEF1Bβ1* protoplasts heated at 42°C. The dark blue line represents the mean granule area at different time points, while the light blue shading indicates the standard error of the mean (SEM). Data are based on the analysis of 6 protoplasts from two independent experiments.
