# Supplementary material for: Developing affordable and efficient heating devices for enhanced live cell imaging in confocal microscopy
Source: Front Plant Sci. 2025 Jan 10;15:1499831. doi: 10.3389/fpls.2024.1499831 (PMC11760603; doi:10.3389/fpls.2024.1499831)
Supplement: Supplementary file 2 [file DataSheet2.pdf]

Supplemental Methods

Protein extraction

Total proteins were extracted from seedlings following a 2-hour treatment at 42°C in a water bath. Plant samples were processed by mixing with a protein extraction buffer in a 3:1 volume-to-weight ratio (100 mM Tris-HCl pH 7.5, 100 mM NaCl, 5 mM EDTA, 5% SDS, 20% glycerol, 20 mM DTT, 40 mM β-mercaptoethanol, 2 mM phenylmethylsulfonyl fluoride, and 0.01% bromophenol blue). The mixture was promptly homogenized using a BeadBug microtube homogenizer (Benchmark Scientific Inc., Sayreville, NJ). The homogenized samples were then boiled for 10 minutes at 100°C, cooled to room temperature, and centrifuged at 21,300 g for 10 minutes. Extracted proteins were stored at -80°C until required for immunoblotting.

Immunoblot analysis

For SDS-PAGE, 8% bis-tris polyacrylamide gels were prepared from 355 mM bis-tris (pH 6.7), 8% acrylamide/bis-acrylamide (37.5:1), 0.07% ammonium persulfate, 0.2% TEMED, and Milli-Q water. Electrophoresis was performed in 1x MOPS buffer at 120V until bromophenol blue reached the gel's bottom. Following electrophoresis, proteins were transferred onto 0.45 µM nitrocellulose membranes (Amersham Protran, MilliporeSigma, Burlington, MA) using a transfer buffer (25 mM Tris Base, 192 mM Glycine, 20% methanol, and Milli-Q water) at 70V for 1 hour. Membranes were rinsed with TBS, blocked for two hours with 2% nonfat milk in TBS, and incubated overnight at 4°C with a rabbit anti-GFP polyclonal antibody (Ab290, Abcam) diluted 1:1000 in TBS, with gentle shaking. Post-primary antibody incubation, membranes were washed with TBS containing 0.05% Tween-20 (TBST), followed by a 30-minute incubation with goat anti-rabbit HRP-conjugated secondary antibody. After extensive washing with TBST, protein bands were visualized using SuperSignal West Pico PLUS Chemiluminescent Substrate (Thermo Fisher Scientific, Waltham, MA) and imaged on an Azure C600 Advanced Imaging System (Azure Biosystems, Dublin, CA). Band intensities were quantified using FIJI/ImageJ software (NIH).
