# Supplementary material for: Developing affordable and efficient heating devices for enhanced live cell imaging in confocal microscopy
Source: Front Plant Sci. 2025 Jan 10;15:1499831. doi: 10.3389/fpls.2024.1499831 (PMC11760603; doi:10.3389/fpls.2024.1499831)
Supplement: Supplementary file 3 [file Table1.docx]

**Supplemental Table S1.** List of materials for construction of one heat plate with associated price guides.

| **Components** | **Source** | **Price** |
| --- | --- | --- |
| Polyimide flexible heater | Omega Engineering Inc., Norwalk, CT, USA | $62.89 |
| Adjustable DC converter transformer | SHNITPWR, Hong Kong, China | $16.55 |
| DC 12V 1-relay temperature controller^1^ | Bayite Technology Co., Shenzhen, China | $20.98 |
| Transparent epoxy resin | Janchun Technology Co., Shenzhen, China | $9.99 |
| Aluminum heat plate (6.3 in x 4.4 in x 0.875 in) | Midwest Steel & Aluminum, Rogers, MN, USA | $49.72^2^ |
| Total | | $160.13 |

^1^This is the PID (Proportional–Integral–Derivative) controller with an integrated thermocouple.

^2^This price does not include the service charge for crafting the aluminum heat plate by the Department of Physics and Astronomy machine shop at the University of Mississippi, University, MS, USA
