# Supplementary material for: Developing affordable and efficient heating devices for enhanced live cell imaging in confocal microscopy
Source: Front Plant Sci. 2025 Jan 10;15:1499831. doi: 10.3389/fpls.2024.1499831 (PMC11760603; doi:10.3389/fpls.2024.1499831)
Supplement: Supplementary file 4 [file Table2.docx]

**Supplemental Table S2.** List of materials for construction of one wireless heater with associated price guides.

| **Components** | **Source** | **Price** |
| --- | --- | --- |
| OMEGA CN32PT-330 PID | Omega Engineering Inc., Norwalk, CT, USA | $240.20 |
| Thermocouple 5TC-TT-K-40-72 type K, gauge 40 | Omega Engineering Inc., Norwalk, CT, USA | $18.90 |
| PID power cable | Bergen Industries Inc., New Berlin, WI, USA | $5.18 |
| Electric wires | Bojack Electron, Dongguan Yuhang Electronic Technology Co., Ltd., China | $3.20 |
| Power supply (12V, 600W) | GESD Pleasure, Amazon | $35.98 |
| Glass slide | Fisher Scientific International, Inc., Hampton, NH, USA | $0.29 |
| Copper foil tape | Lovimag, Nanjing Anshiqing E-commerce Co., Ltd, China | $1.99 |
| Cable connector | Romeda, Amazon | $2.39 |
| Charging coil (12V, 2.5A) | Taidacent, Shenzhen Taida Century Technology Co., Ltd., China | $18.00 |
| Total | | $326.13 |
