# Supplementary material for: Developing affordable and efficient heating devices for enhanced live cell imaging in confocal microscopy
Source: Front Plant Sci. 2025 Jan 10;15:1499831. doi: 10.3389/fpls.2024.1499831 (PMC11760603; doi:10.3389/fpls.2024.1499831)
Supplement: Supplementary file 5 [file Table3.docx]

**Supplemental Table S3.** Primers used for plasmid construction.

| **Fragment** | **Forward primer** | **Reverse primer** | **Note** |
| --- | --- | --- | --- |
| AtHSP70-4pro | AGCTATGACCATGATTACGatcaatccgttcttttagcagtg | CGTATGGGTAAACGGCCATTATTAGAGATCAGAATTGTTCGCC | To clone 1362-bp promoter sequence of *AtHSP70-4* |
| 3×HA | ATGGCCGTTTACCCATACGATG | CGGAGCGTAATCTGGAACG | To clone the *3×HA* tag |
| YFP-(PT)_4_P | CGTTCCAGATTACGCTCCGATGGTGAGCAAGGGCGAG | AGGGGTGGGAGTTGGTGTTGGAGTAGGCTTGTACAGCTCGTCCATGC | To clone *YFP* with the *(PT)_4_P* linker |
| AtHSP70-4 CDS | AACACCAACTCCCACCCCTATGGCGGGTAAAGGTGAAG | CTGCAGGTCGACTCTAGAGTTAATCAACTTCTTCAATCTTTGGG | To clone the coding sequence of *AtHSP70-4* |
| All fragments were cloned into *pJHA212H* between EcoRI and BamHI using NEB HiFi assembly to construct the *pJHA212H/AtHSP70-4pro:: 3×HA-YFP-(PT)_4_P-AtHSP70-4* plasmid. | | | |
